# Suppression of plastid-to-nucleus gene transfer by DNA double-strand break repair

**DOI:** 10.1038/s41477-025-02005-w

**Published:** 2025-05-16

**Authors:** Enrique Gonzalez-Duran, Xenia Kroop, Anne Schadach, Ralph Bock

**Affiliations:** Max-Planck-Institut für Molekulare Pflanzenphysiologie, Potsdam-Golm, Germany

**Keywords:** Plant genetics, Evolution, Chloroplasts, Plant evolution, Genome evolution

## Abstract

Plant nuclear genomes contain thousands of genes of mitochondrial and plastid origin as the result of endosymbiotic gene transfer (EGT). EGT is a still-ongoing process, but the molecular mechanisms determining its frequency remain largely unknown. Here we demonstrate that nuclear double-strand break (DSB) repair is a strong suppressor of EGT. Through large-scale genetic screens in tobacco plants, we found that EGT from plastids to the nucleus occurs more frequently in somatic cells when individual DSB repair pathways are inactive. This effect is explained by the expected increase in the number and residence time of DSBs available as integration sites for organellar DNA. We also show that impaired DSB repair causes EGT to increase 5- to 20-fold in the male gametophyte. Together, our data (1) uncover DSB levels as a key determinant of EGT frequency, (2) reveal the strong mutagenic potential of organellar DNA and (3) suggest that changes in DNA repair capacity can impact EGT across evolutionary timescales.

## Abstract (web summary)

Inactivation of double-strand break repair pathways greatly increases the integration of plastid DNA into the nuclear genome of tobacco plants, highlighting the mutagenic potential of organellar DNA and its role in shaping plant nuclear genomes.

## Main

During the evolution of eukaryotes, genes originally encoded in the genomes of mitochondria and plastids (chloroplasts) have been relocated to the nuclear genome through endosymbiotic gene transfer (EGT). EGT enables the evolutionary transition of symbionts to organelles, including the transition of the cyanobacterial endosymbiont to the plastids of photosynthetic eukaryotes (algae and plants). The elucidation of the molecular mechanisms controlling EGT is of critical importance to our understanding of eukaryotic and plant evolution.

EGT, also known as intracellular gene transfer, is believed to occur largely through events of organelle rupture that release organellar DNA (orgDNA) into the cytosol, thus resulting in a flow of genetic information towards the nucleus over evolutionary time^1,2^. As a consequence, nuclear genomes contain thousands of genes that are of mitochondrial or chloroplast origin, and they also harbour so-called promiscuous orgDNA sequences that originate from recent transfer events from mitochondria (nuclear mitochondrial sequences)^3^ or plastids (nuclear plastid sequences (NUPTs)), collectively referred to as nuclear organellar sequences (NORGs). While NORGs are often non-functional, the coding sequences contained therein can become functional in the nucleus by acquiring suitable (eukaryotic-type) expression elements and signals for subcellular targeting. The relocation of genes from the organellar to the nuclear genome may entail substantial evolutionary pay-offs, including access to sexual recombination^4,5^ and more energy-efficient gene maintenance^6^. NORG insertion can also influence the composition of the nuclear genome. In plants, NORGs usually comprise between 0.1% and 1% of the nuclear genome^7^, but much higher values have also been observed. For example, in *Moringa oleifera*, a fast-growing, deciduous tree in the order Brassicales, NUPTs alone account for 4.7% of the nuclear genome^8^. Thus, variations in the frequency and genetic control of EGT can substantially affect plant genome size and evolution.

NORGs arise through a three-step mechanism: (1) the escape of DNA from the organelle, (2) the translocation of the released orgDNA to the nucleus and (3) integration into the nuclear genome. In yeast (*Saccharomyces cerevisiae*), a DNA plasmid can be transferred from the mitochondrion to the nucleus, where it is maintained episomally^9^. Subsequent genetic screens found mutants (*yme1*–*yme6*) with increased frequencies of mitochondrial plasmid DNA transfer to the nucleus^10^. The mutated genes^11–14^ are probably involved in mitochondrial biogenesis, degradation and/or integrity, suggesting that the escape rate of DNA from the mitochondrion may limit EGT^15,16^.

The genetic control of the integration of the released DNA into the nuclear genome is not understood. While the integration of short mitochondrial DNA fragments (<200 bp) has been observed in yeast^17^, the integration of functional EGT reporters has been achieved only for plastid DNA in tobacco (*Nicotiana tabacum*)^18–20^. In this system, NUPTs arise from the transfer and integration of genomic plastid DNA into the nucleus, often as large fragments (>20 kb)^21,22^. The frequency of plastid DNA translocation to the nucleus is much higher than that of plastid DNA integration into the nuclear genome^23^, suggesting integration efficiency as a bottleneck of EGT.

In eukaryotes, the integration of DNA into the nuclear genome requires the activity of conserved pathways that repair double-strand breaks (DSBs)^24–26^. In the model plant *Arabidopsis thaliana*, somatic DSBs are repaired nearly exclusively by non-homologous end joining (NHEJ) or microhomology-mediated end joining (MMEJ)^27^, whereas repair by homologous recombination (HR) is very rare^28^. Recently, NHEJ has been implicated in NORG integration in the apicomplexan *Toxoplasma gondii*^29^, whereas in plants, sequence analyses of natural^30–32^ and experimentally generated NORGs^21,33^ suggest that both NHEJ and MMEJ participate in EGT. However, it is unknown whether and to what extent these pathways control the frequency of EGT. Also, no genes have been identified that would affect EGT frequency in plants or other multicellular eukaryotes. To obtain insights into the genetic control of EGT, we set out to study how DSB repair (DSBR) pathways control EGT in plants, using tobacco as a model species.

## Results

### Generation of DSBR mutants

**Fig. 1:**
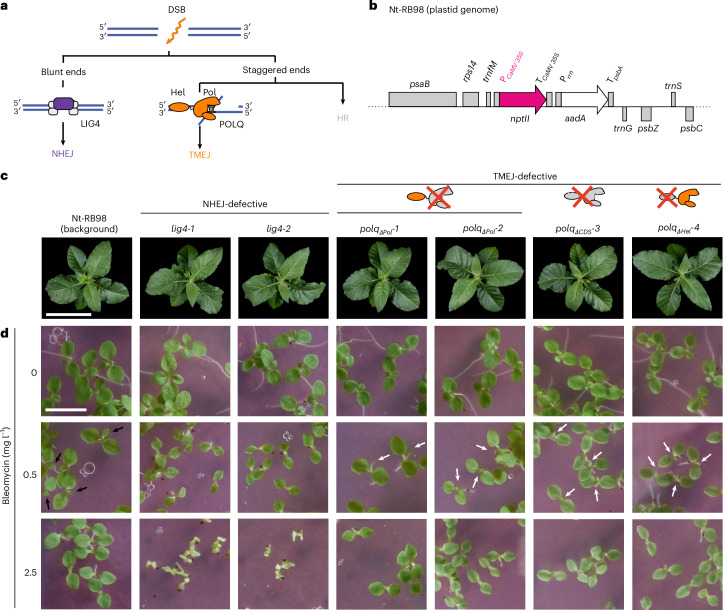
Generation of mutants defective in DSBR in *N. tabacum.* **a**, The main pathways of DSBR in eukaryotes. LIG4 and POLQ are the main factors acting in NHEJ and TMEJ, respectively. **b**, Physical map of the engineered region in the plastid genome of Nt-RB98 plants containing an EGT reporter between the *trnfM* and *trnG* genes. *nptII* is a kanamycin resistance marker under the control of the strong nuclear CaMV (cauliflower mosaic virus) 35S promoter. Cells can become kanamycin resistant only upon translocation of this cassette to the nucleus. The *aadA* gene is under the control of the strong plastid promoter *P*_*rrn*_ and confers constitutive resistance to spectinomycin. **c**, Plants after 45 days of growth in sterile culture followed by 24 days of growth in soil. The images are representative of three independent cultivation experiments. Scale bar, 20 cm. **d**, Seedlings germinated in medium containing bleomycin (a DSB-generating drug) at 17 days after sowing. At 0.5 mg l^−1^, bleomycin sensitivity is evidenced by pointed cotyledons and the lack of true leaves in the *lig4* mutants, whereas in *polq* mutants, the emerging true leaves (white arrows) are narrow and stunted compared with the Nt-RB98 control (black arrows). At 2.5 mg l^−1^ bleomycin, *lig4* mutants fail to grow, and *polq* mutants display pointed cotyledons. The images are representative of two independent experiments. Scale bar, 1 cm.

To determine whether DSBR pathways control EGT in plants, we generated loss-of-function mutants of the tobacco homologues of DNA ligase IV (LIG4), an essential component of the NHEJ pathway^34–36^, and of DNA polymerase theta (Polθ or POLQ), which is required for polymerase-theta-mediated MMEJ (or TMEJ)^37–40^ (Fig. 1a). LIG4 and POLQ are suitable targets in that they (1) are necessary for the function of their respective DSBR pathways, (2) have not been described as required for any other DSBR pathway and/or for unrelated cellular functions, and (3) are not part of large gene families, thus allowing the unambiguous identification of their orthologues in tobacco.

**Extended Data Fig. 1:**
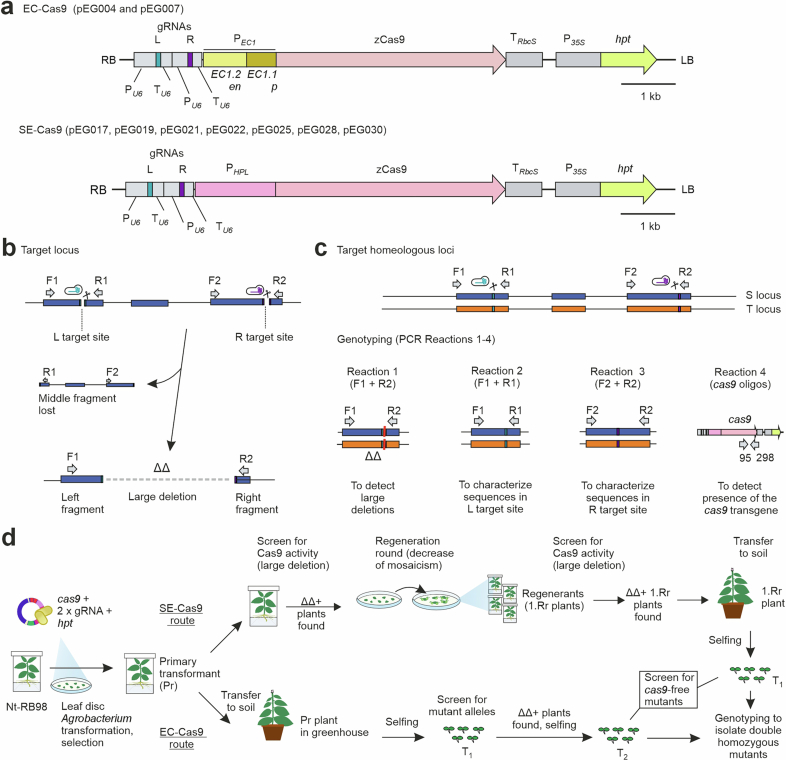
Strategy for the generation of DSBR mutants in *N. tabacum.* **a**. Constructs (T-DNA) for *Agrobacterium*-mediated transformation of tobacco, either for expression of Cas9 in the egg cell (EC-Cas9) or for constitutive somatic expression (SE-Cas9). *EC1.2en* and *EC1.1p* are the enhancer and promoter, respectively, of the *EC1* gene from *Arabidopsis thaliana* (AT2G21740). P_U6_ and T_U6_ are promoter and terminator, respectively, of the U6 snRNA gene from *A. thaliana*. gRNAs L and R are represented by cyan and purple boxes, respectively. P_*HPL*_ is the promoter from the *HPL* gene of *A. thaliana* (AT4G15440). zCas9 is a *cas9* gene version that was codon-optimized for *Zea mays*. T_RbcS_, Rubisco small subunit terminator; P_35S_, double CaMV 35S promoter; *hpt*, hygromycin phosphotransferase gene conferring resistance to hygromycin *in planta*. **b**. Detection of Cas9 activity is based on the generation of a large deletion (ΔΔ) between the cut sites of gRNA L and R using primers F1 and R2 (grey arrows) **c**. Genotyping strategy based on PCR. Reactions 1 to 3 are defined in relation to the gRNA target sites. Reaction type 4 (oligos oEG95 and oEG298) was used to detect the presence of the *cas9* transgene. **d**. Transformation and screening strategy relying on the detection of large deletion alleles (ΔΔ+) by Reaction 1. For SE-Cas9 transformants, the detection of alleles was conducted during vegetative plant propagation in the T_0_ generation with Reactions 1-3, whereas EC-Cas9 mutagenesis requires an egg-cell passage, and consequently, plants were screened in T_1_. Rr: regeneration round.

Mutants were generated by CRISPR–Cas9-mediated genome editing using two alternative strategies that relied on Cas9 expression in mesophyll cells^41^ or in the egg cell (Extended Data Fig. 1). To enable subsequent genetic screens for EGT, all mutants were generated in the transplastomic Nt-RB98 background, whose plastid genome contains an *nptII* cassette under the control of a strong nuclear promoter that confers kanamycin resistance only upon transfer to the nucleus^18^ (Fig. 1b).

**Extended Data Fig. 2:**
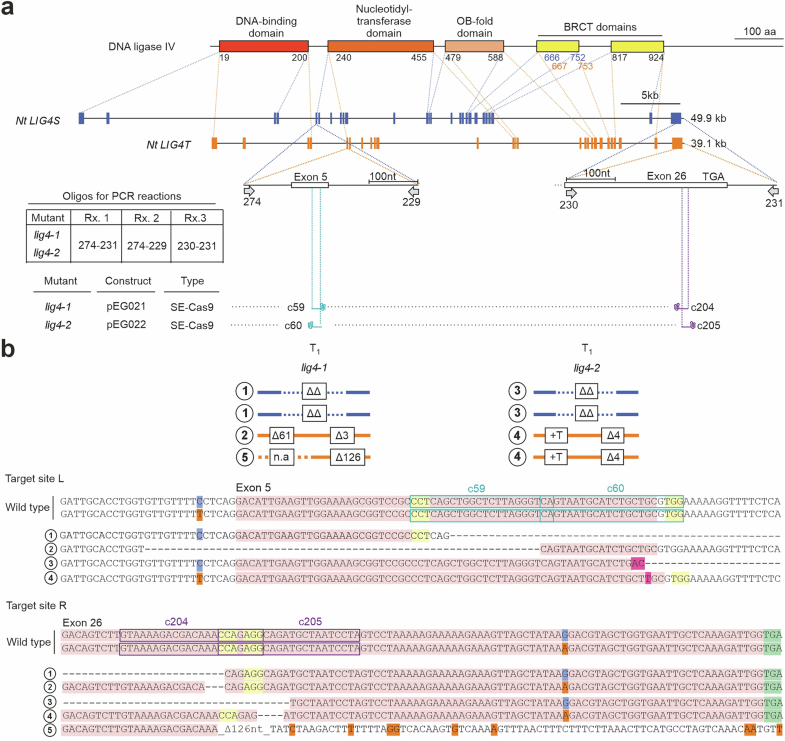
Generation of mutants in tobacco *LIG4* homologs. **a**. *LIG4* homologs in *N. tabacum*. Top: domain structure of DNA ligase IV proteins in tobacco. Boxes represent functional domains, and the amino acid positions marking their borders are given. Middle: locus structures at the genomic level. Boxes represent the exons. Arrows represent PCR primers, and the numbers denote oligonucleotides of the oEGxxx series (Supplementary Table 10). The native stop codon (TGA) is indicated. The table in the left shows the combinations of primer pairs for genotyping reactions (Rx.) according to the types defined in Extended Data Fig. 1c. Bottom: Pairs of gRNAs L (cyan) and R (purple) for genome editing of *LIG4* homologs. **b**. Genomic sequences of *lig4* mutants. Top: schematic representations of the mutant genotypes. White boxes represent mutations in the L site (left), R site (right) or large deletions (ΔΔ) spanning both sites (centered) in the homologous alleles (blue and orange, respectively) from the two diploid progenitor species that formed the allotetraploid *N. tabacum*. n.a: not amplifiable by PCR. Bottom: *LIG4* genomic sequences in the mutants obtained by Sanger sequencing. Coding sequences are shaded in pink; polymorphisms between the two loci are shaded in blue (*N. sylvestris* locus; S) and orange (*N. tomentosiformis* locus; T), respectively. Insertions are shaded in magenta, and native stop codons in green. gRNA target sequences are boxed (in cyan or purple), and the protospacer adjacent motif (PAM) is marked by yellow shading.

**Extended Data Fig. 3:**
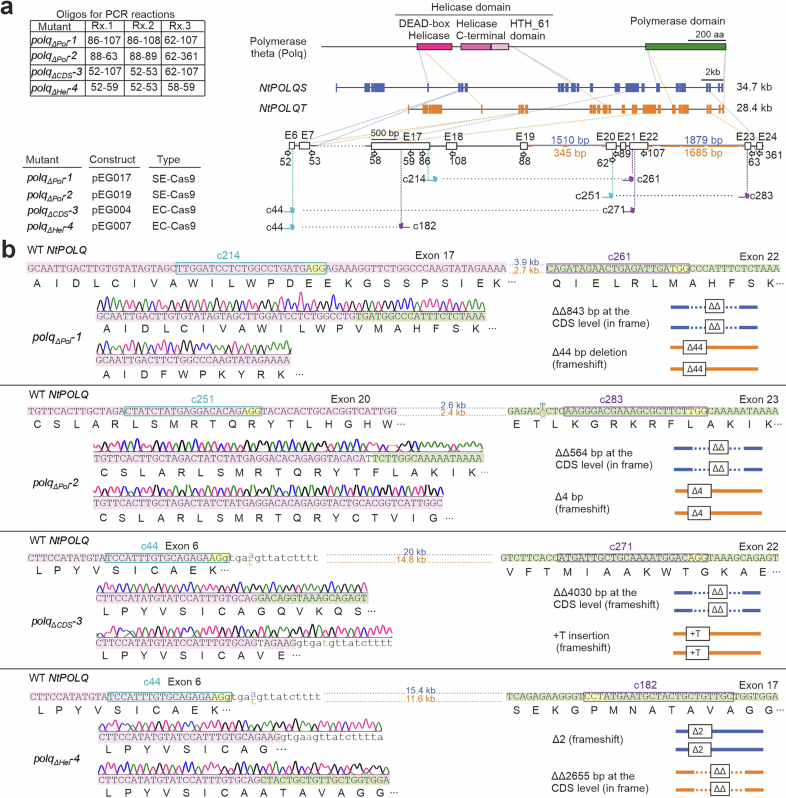
Generation of mutants in tobacco *POLQ* homologs. **a**. *POLQ* homologs in *N. tabacum*. Top right: domain structure of the POLQ proteins in tobacco. Boxes represent functional domains, and the amino acid positions at their borders are indicated. Middle right: locus structures at the genomic level. Boxes represent exons, arrows mark binding sites for PCR primers (with the numbers denoting oligonucleotides of the oEGxxx series; Supplementary Table 10). Top left: combinations of primer pairs for genotyping reactions (Rx.) according to the types defined in Extended Data Fig. 1c. Bottom right: Pairs of gRNAs L (cyan) and R (purple) for genome editing. **b**. Genomic sequences of *polq* mutants. Coding sequences are in uppercase letters and are shaded in pink or green; sequences in lowercase letters indicate introns. Polymorphisms and differences in genomic distances between the S and T wild-type alleles (WT) are shown in blue and orange letters, respectively. gRNA target sequences are boxed (in cyan or purple). The protospacer adjacent motif (PAM) is shaded in yellow. A schematic representation of the *polq* alleles is shown for each mutant at the right. S and T homeoalleles are represented by blue and orange, respectively. The mutation types are indicated in white boxes, with boxes positioned within the left half of the gene representing mutations at the L site, and centered boxes indicating large deletion mutations between the L and R sites.

The genome of tobacco, an allotetraploid^42^, contains two loci for each of the target genes, thus necessitating the generation of double knockouts (Extended Data Fig. 1). Using different constructs, we produced two independent *lig4* knockout lines (*lig4-1* and *lig4-2*; Extended Data Fig. 2). The POLQ protein contains a helicase and a polymerase domain, and while both are necessary for efficient MMEJ^43,44^, the helicase domain has additional functions^45^ and may participate in safeguarding genome stability^46,47^. With different knockout constructs, we succeeded in generating four independent doubly homozygous *polq* mutants (*polq*_*ΔPol*_*-1*, *polq*_*ΔPol*_*-2*, *polq*_*ΔCDS*_*-3* and *polq*_*ΔHel*_*-4*; Extended Data Fig. 3). In these mutants, POLQ is knocked out at one locus by a (homozygous) frameshift mutation, while the other locus harbours in-frame deletions that remove the polymerase domain (ΔPol) or the helicase domain (ΔHel), or alternatively a large deletion comprising both domains (ΔCDS). The helicase-defective mutants (*polq*_*ΔCDS*_*-3* and *polq*_*ΔHel*_*-4*) were obtained only after expressing Cas9 in the egg cell, and all attempts to generate them through Cas9 expression in the mesophyll produced only mosaic plants. The *cas9* transgene was segregated out from all *lig4* and *polq* mutants prior to their use in further experiments.

**Extended Data Fig. 4:**
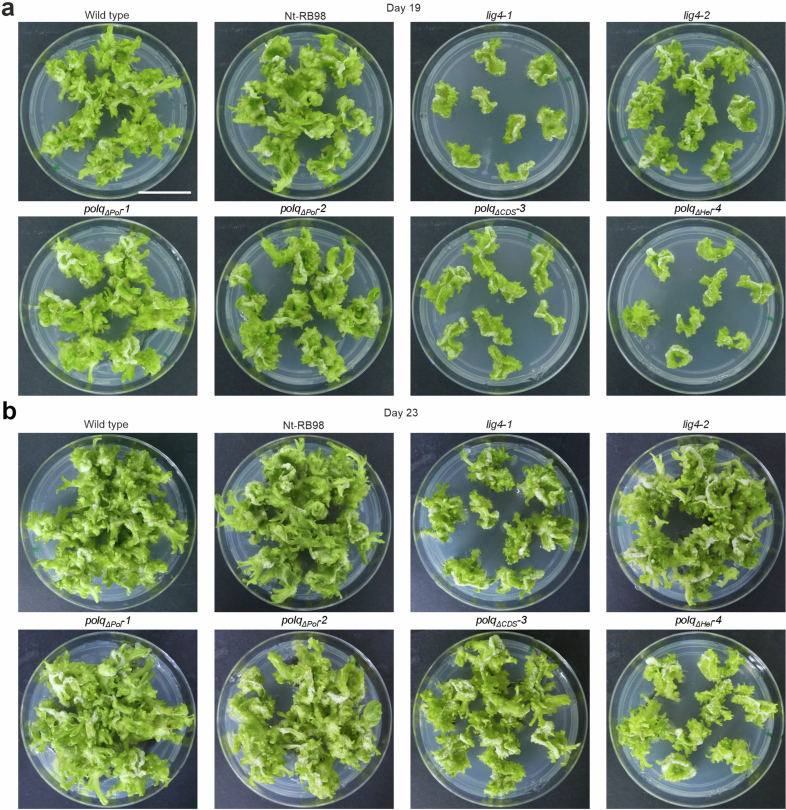
Regeneration capacity of DSBR mutants in tissue culture. Tobacco explants at 19 days **(a)** and 23 days (**b**) after transfer to regeneration medium without antibiotics. *polq*_*ΔCDS*_*-3* and *polq*_*ΔHel*_*-4* display a delay in shoot regeneration. A similar delay is seen in *lig4-1*, but is absent from *lig4-2*. Despite the delay in the onset of regeneration, all genotypes overgrow the plates by day 30. Scale bar: 3 cm.

The *lig4* and *polq* mutants show normal vegetative development in standard growth conditions (Fig. 1c) and readily regenerate shoots in tissue culture (Extended Data Fig. 4). Relative to Nt-RB98, regeneration is slightly slower in *polq*_*ΔCDS*_*-3* and *polq*_*ΔHel*_*-4*, but not in *polq*_*ΔPol*_*-1* or *polq*_*ΔPol*_*-2*. This phenotypic difference, together with the difficult mutant isolation, could be explained by a role of POLQ in replicative stress tolerance during vegetative growth, which was previously reported in *Arabidopsis*^48^. However, our results strongly suggest that the role of POLQ in replicative stress tolerance is specific to its helicase domain and independent of the role of POLQ in TMEJ (which requires the polymerase domain).

**Extended Data Fig. 5:**
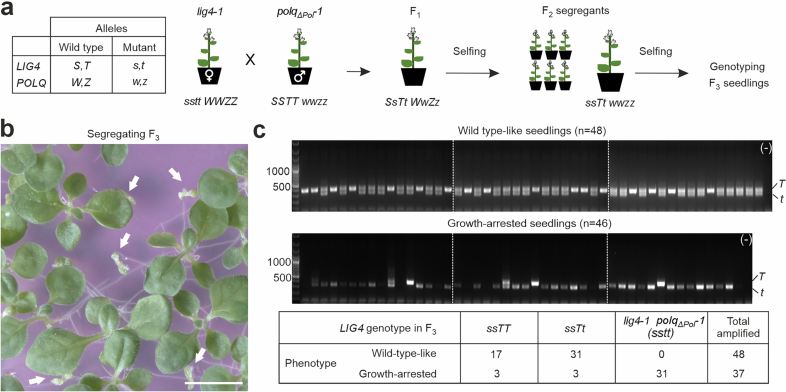
Phenotype of a tobacco mutant simultaneously defective in NHEJ and TMEJ. **a**. Strategy for the isolation of *lig4-1 polq*_ΔPol_*-1* quadruple mutants. *s* and *t* correspond to the two alleles present in a double homozygous derivative of *lig4-1* (the large deletion and the Δ61 allele at the *S* and *T* loci, respectively; Extended Data Fig. 2); *w* and *z* are the mutant alleles of *polq*_ΔPol_*-1* (Extended Data Fig. 3). After genotyping of 96 seedlings in the F_2_ generation, we obtained a *polq*_ΔPol_*-1* mutant that was homozygous for the large deletion in the *LIG4S* locus and heterozygous in the *LIG4T* locus. **b**. Segregating F_3_ seedlings 28 days after sowing. Some seedlings (white arrows) show delayed germination, growth arrest and eventual lethality at the seedling stage. Scale bar: 1 cm **c**. Genotyping of the *LIG4* loci in F_3_ seedlings. PCR using primers oEG274 and oEG229 targets the region flanking the L site and simultaneously amplifies the alleles *T* and *t*. The right lane contains a negative control (-) with water added instead of genomic DNA. Samples where only the lower band (*t* allele) is amplified represent *lig4-1 polqpol-1* quadruple mutants. They correspond to the growth-arrested seedlings. Source data

To confirm that the generated mutants are defective in DSBR, we examined seedling development in the presence of the DSB-generating drug bleomycin. All mutants displayed higher bleomycin sensitivity than the control line Nt-RB98 (Fig. 1d), confirming their DSBR deficiency. The *lig4* mutants were more severely affected than the *polq* mutants, indicating that NHEJ may be more active in DSBR than TMEJ. Moreover, all *polq* mutants displayed similar bleomycin sensitivity, suggesting that disruption of either the helicase or the polymerase domain is sufficient to knock out TMEJ. Our attempts to generate a *lig4-1* *polq*_*ΔPol*_*-1* quadruple mutant through crossing revealed that this genotype is seedling-lethal (Extended Data Fig. 5), demonstrating that the combined loss of NHEJ and TMEJ cannot be compensated by other repair pathways in tobacco.

**Extended Data Fig. 6:**
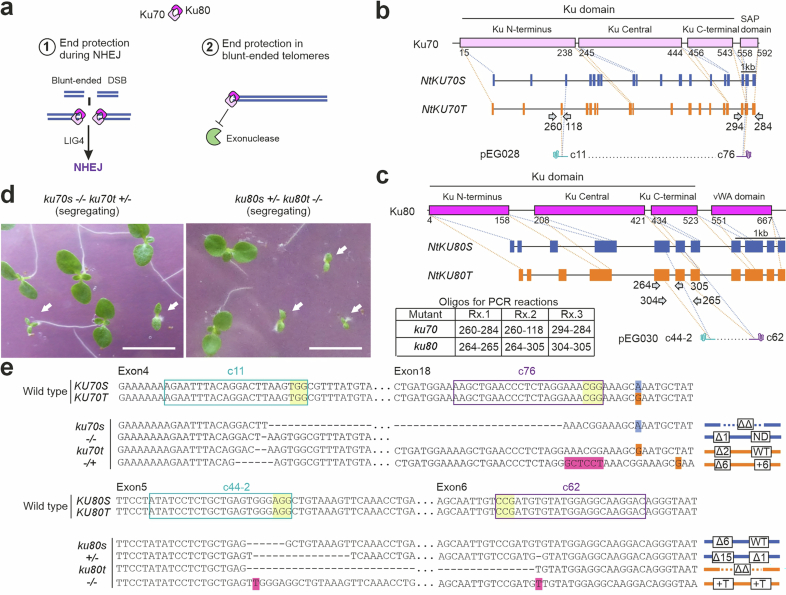
Generation of mutants in tobacco *KU70* and *KU80* homologs. **a**. The Ku heterodimer binds dsDNA ends to protect them from nucleolytic resection. **b-c**. Homologs of *KU70* (**b**) and *KU80* (**c**) in *N. tabacum*. Top: domain structure at the protein level. Boxes represent functional domains, and amino acid positions marking their borders are given. Middle: locus structures at the genomic level. Boxes represent the exons. Arrows represent PCR primers, and the numbers denote oligonucleotides of the oEGxxx series (Supplementary Table 10). Bottom: Pairs of gRNAs L (cyan) and R (purple) for genome editing. Bottom left corner: the combinations of primer pairs for genotyping reactions (Rx.) according to the types defined in Extended Data Fig. 1c. **d**. Progeny of heterozygous *ku70* (left) and *ku80* (right) mutants 14 days after sowing. Segregation produces seedlings displaying strong growth arrest (white arrows), which harbor only frameshift and/or large deletion alleles (n = 12 and 13 analyzed, respectively), as well as wild-type-like seedlings that harbor at least one allele that retains the native reading frame (*n* = 32 and 48 analyzed, respectively). Scale bars: 1 cm. **e**. Genomic sequences of the heterozygous *ku* mutants. Left: genomic sequences in the *ku* mutants obtained by Sanger sequencing. Polymorphisms between the two loci are shaded in blue (*N. sylvestris* locus; S) and orange (*N. tomentosiformis* locus; T), respectively. Insertions are shaded in magenta. gRNA target sequences are boxed (in cyan or purple), including the protospacer adjacent motif (PAM; shaded in yellow). Right: schematic representations of the mutant genotypes. White boxes represent mutations in the L site (left), R site (right) or large deletions (ΔΔ) spanning both sites (centered) in the homologous alleles (blue and orange, respectively) from the two diploid progenitor species of the allotetraploid species *N. tabacum*. WT: wild-type sequence. ND: not determined.

Despite the minor role of HR as a DSBR pathway in somatic cells of seed plants, we attempted to generate an HR-defective mutant by targeting the *RPA1C* gene, encoding a subunit of the RPA (replication protein A) heterotrimer^49^. The phenotypes of *Arabidopsis* mutants lacking functional RPA1C-family proteins^50^ suggest that they might have a specific defect in somatic HR. However, our efforts to isolate a tobacco *rpa1c* mutant by genome editing were unsuccessful in that only chimeric plants could be recovered. We also attempted to generate additional NHEJ-defective mutants by targeting Ku70 and Ku80 (Extended Data Fig. 6). While we were able to generate stable heterozygous mutant lines, the homozygous knockout seedlings displayed strong growth arrest and altered morphology, which rendered them unsuitable for use in EGT screens. These strong mutant phenotypes are not entirely surprising, given that Ku70 and Ku80 have functions in telomere homeostasis^51–53^ in addition to NHEJ, and in rice (*Oryza sativa*), the disruption of Ku also causes developmental defects^54,55^.

### Defects in DSBR strongly increase somatic EGT

**Fig. 2:**
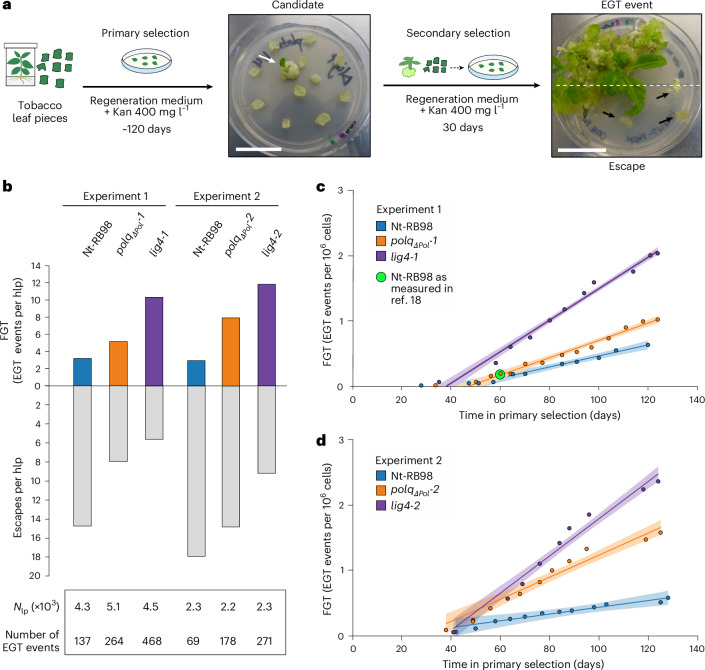
DSBR deficiency causes increased EGT in somatic cells. **a**, Workflow of the genetic screens for EGT based on kanamycin (Kan) selection. Plants regenerating during primary selection (white arrow) are considered candidate EGT lines. Lines that also regenerate during secondary selection are considered genuine EGT events, while lines that are kanamycin sensitive during secondary selection (black arrows) are considered ‘escapes’. Scale bars, 3 cm. **b**, Frequencies of gene transfer events (coloured) and escapes (grey) per hundred leaf pieces (hlp) in each genotype at the end of Experiments 1 and 2. *N*_lp_, number of leaf pieces screened. **c**,**d**, Change in the cumulative FGT during primary selection in Experiments 1 (**c**) and 2 (**d**). The calculations of FGT per cell number are based on previous estimates of cell counts in tobacco leaves (Methods). The change of FGT over time per genotype was analysed through simultaneous linear regressions (Models 1 and 2, Supplementary Table 1). Each data point corresponds to an FGT calculated using the total number of EGT events that had been obtained up to that day (cumulative FGT). The coloured shading corresponds to the 95% confidence interval of the cumulative FGT, calculated according to the values fitted by Models 1 and 2 at each time point. The filled green circle represents a single FGT value for Nt-RB98 (equivalent to one event per hlp) reported in a previous study^18^, based on a measurement made at the end of a fixed primary selection period of 60 days (in an otherwise similar experimental set-up; the time dependence of FGT measurements was unknown at the time)^18^.

We first investigated the effect of DSBR deficiency on EGT in somatic cells in two independent large-scale experiments. To screen for EGT events, leaf explants from plants containing the plastid-encoded EGT reporter construct RB98 (*lig4* mutants, *polq* mutants and control plants with a wild-type nuclear background) were placed onto regeneration medium containing kanamycin for ~120 days (‘primary selection’; *n* = 20,813 leaf pieces; ~1.04 billion cells). Regenerants from primary selection were considered candidate events of *nptII* transfer to the nucleus (Fig. 2a). Candidates were transferred to fresh medium for a secondary selection step. Persistent kanamycin resistance was indicative of *nptII* transfer and stable integration into the nuclear genome (‘EGT events’), whereas sensitive lines were considered false positives (‘escapes’) at this stage. From the counts of EGT events, we calculated a frequency of gene transfer (FGT) that is normalized to the amount of screened material and expressed either as EGT events per hundred leaf pieces or as EGT events per number of cells.

After the completion of the two selection steps, it became apparent that FGTs are significantly higher in the DSBR mutants than in the wild-type nuclear background (line Nt-RB98; Fig. 2b), demonstrating that deficient DSBR promotes EGT in somatic cells. This finding, together with the seedling-lethal phenotype of the NHEJ/TMEJ double knockout, suggests that NHEJ and TMEJ control EGT through a dual relationship: on the one hand, the pathways act as EGT suppressors (when both of them are active), and, on the other hand, it is very likely that at least one of these pathways is required for orgDNA integration in EGT to occur.

To determine whether our FGT measurements had been impacted by the length of the primary selection phase, we analysed the changes in cumulative FGT over time. Surprisingly, we found that the cumulative FGT increases linearly over time in all genotypes but grows more rapidly in the DSBR mutants (Fig. 2c,d and Supplementary Tables 1 and 2). Consequently, FGT measurements are strongly time-dependent, in that the observed frequencies change over time at a genotype-specific, relatively constant rate, which we define here as the gene transfer rate (GTR, expressed in EGT events per cell per day). The DSBR mutants have significantly higher GTRs than the Nt-RB98 control plants, and in the *lig4-2* mutant, the GTR is one event per 35 million cells per day (Supplementary Table 1). Clearly, such a high rate will cause a substantial number of somatic mutations during the lifespan of the organism.

### Deficient DSBR increases DNA integration

The discovery of constant GTRs contradicted our initial expectation that the cumulative FGT would be high at early time points and then reach saturation. This would have indicated that most EGT events occurred prior to the selection experiment. Instead, our results suggest that EGT events also occur in real time (that is, during primary selection). This is probably possible because inhibitors of chloroplast translation such as kanamycin are known to confer non-lethal selection, in that they allow cells to survive for long periods in the presence of the antibiotic^56^. The leaf explants thus continuously produce EGT events that we measure in our screens.

**Fig. 3:**
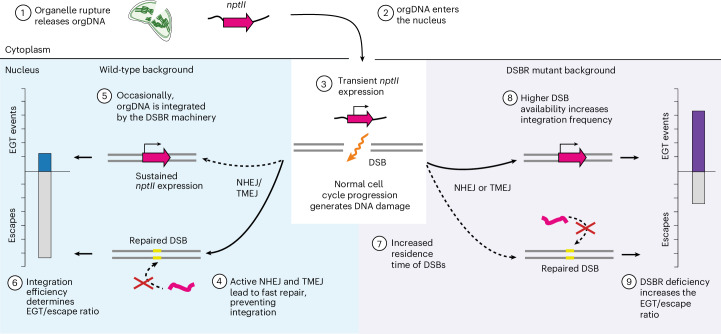
Molecular mechanisms involved in nuclear integration of escaped orgDNA during EGT. In our experimental design, the entry into the nucleus of a functional *nptII* gene leads to kanamycin detoxification and enables outgrowth of a candidate EGT event during primary selection, regardless of whether the orgDNA has integrated into the nuclear genome. Resistance in secondary selection depends on whether the *nptII* integrates in the nuclear genome. Upon integration, *nptII* maintenance and expression become coupled to cell division and result in sustained kanamycin resistance, facilitating regeneration in secondary selection. Failure to integrate results in the loss of the escaped *nptII* gene from the regenerating tissue, thus leading to kanamycin sensitivity in secondary selection. Reduced DSBR capacity increases the number of DSBs available for the integration of orgDNA, which is catalysed by the remaining active pathway(s). This increases the success rate of orgDNA integration, which is reflected by an increased recovery of EGT events versus escapes during secondary selection.

Our genetic screens uncovered an inverse relationship between EGT events and escapes produced by each genotype (Fig. 2b). This finding suggests that what we initially had classified as escapes are in fact non-productive (that is, transient) gene transfer events (Fig. 3), in which stable integration of the escaped *nptII* into the nuclear DNA did not occur. Consequently, both stable EGT events and ‘escape’ events are the products of *nptII* translocation to the nucleus: whereas stable EGT events result from the integration of *nptII* into the nuclear genome, ‘escapes’ result from *nptII* transfer events without integration (Fig. 3). Consequently, the proportion of EGT lines to total candidate lines in each genotype reflects the success rate of orgDNA integration into the nucleus. The elevated frequencies of stable EGT in the repair-deficient mutants can be explained by the increased residence time of nuclear DSBs, which provides more opportunities for orgDNA integration. The number of DSBs at a given time is presumably proportional to the extent of DSBR impairment, which appears to be stronger in NHEJ-defective mutants than in TMEJ-defective mutants (Figs. 1d and 2b–d). As expected, the total numbers of candidate lines (stable + transient events) obtained for all genotypes are very similar within each experiment (Fig. 2b and Supplementary Table 2) and probably reflect the unaltered rates of organelle rupture and orgDNA release into the cytosol (Fig. 3). This observation is consistent with our experience with kanamycin selection experiments that use high concentrations of the antibiotic, which suggest that the strength of selection remains stable for over 120 days (in that transgenic lines continue to be recovered, and no background regeneration occurs).

### DSBR pathways influence NORG stability

We obtained seeds from 871 fertile plant lines derived from somatic EGT events obtained in our screens (Supplementary Table 3). In their progenies, the kanamycin resistance trait is expected to display Mendelian segregation (that is, 1:1 when crossed with the wild type), if gene transfer results in a single nuclear *nptII* locus (Supplementary Table 4). Approximately one third of the EGT lines displayed lower-than-expected segregation ratios, which, in principle, could be attributable to instability of the *nptII* locus in the nucleus (a common phenomenon in EGT experiments^57^), or alternatively to gene silencing of the escaped *nptII*^58^. Genotyping for the presence of *nptII* in the progeny of EGT lines from all three genetic backgrounds revealed that kanamycin-sensitive seedlings lack *nptII* in most of the analysed lines with distorted segregation ratios (Supplementary Table 5), suggesting genetic instability (rather than gene silencing) as the main cause of the underrepresentation of kanamycin-resistant seedlings^57^. Surprisingly, transgenerational instability of the kanamycin resistance trait is much less frequent in the progenies of *lig4*-derived EGT lines (8–19% of lines; Supplementary Table 4), indicating that defective NHEJ leads to increased stability and/or improved expression of the transferred gene.

### High-frequency EGT in the male germline of DSBR mutants

**Fig. 4:**
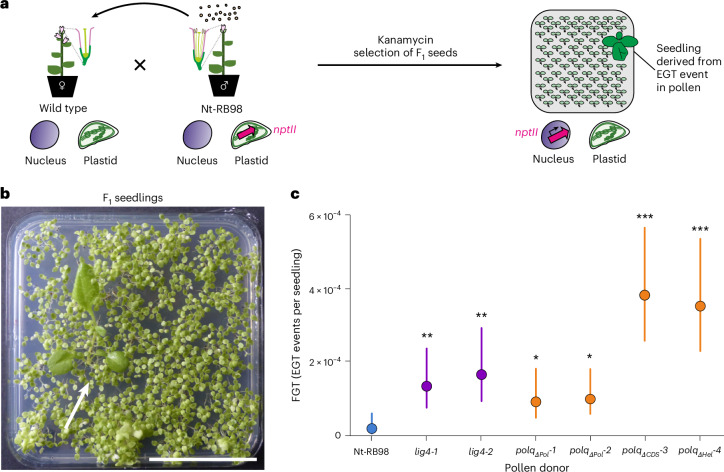
DSBR defects cause strongly increased EGT in the male germline. **a**, Experimental design to measure EGT in the germline. Seedlings derived from the genetic cross shown are kanamycin resistant only if derived from a pollen grain (sperm cell) where an EGT event resulted in *nptII* integration into the nuclear genome. **b**, Screening of the F_1_ progeny of a wild-type tobacco × *polq*_*ΔPol*_*-*1 cross on medium with kanamycin (100 mg l^−1^). The plate was photographed 21 days after sowing. A kanamycin-resistant seedling (white arrow) is readily identified by its dark-green pigmentation, unimpaired growth and development of true leaves. Scale bar, 5 cm. **c**, Quantification of the FGT in the male germline. The effects of the mutant genotypes on FGT compared with the Nt-RB98 control were estimated through a model based on the binomial distribution (*n* = 647,200 seedlings; Model 3; Methods and Supplementary Table 6). The filled circles represent mean FGTs per genotype, calculated on the basis of seedling counts from up to two independent crossing experiments (Supplementary Table 6). The coloured vertical bars represent the 95% confidence intervals according to Model 3. The *P* values result from simultaneous two-tailed Wald *z*-tests of the parameters in the model (each representing a mutant–control comparison) and are adjusted for multiple comparisons using the Holm–Bonferroni method. All comparisons revealed significant differences (*lig4-1*, *P* = 0.00825; *lig4-2*, *P* = 0.00359; *polq*_*ΔPol*_*-1*, *P* = 0.0219; *polq*_*ΔPol*_*-2*, *P* = 0.0219; *polq*_*ΔCDS*_*-3*, *P* = 6.7 × 10^−6^; *polq*_*ΔHel*_*-4*, *P* = 1.3 × 10^−5^). *P < 0.05; **P < 0.01; ***P < 0.001.

EGT occurs much more frequently in the male gametophyte (pollen) than in the female gametophyte or in somatic cells^23^. To determine whether defects in DSBR change the abundance of EGT in pollen, we crossed DSBR mutants as pollen donors to wild-type recipients (Fig. 4a). Kanamycin resistance in the progeny is indicative of EGT events that probably occurred during pollen development (Fig. 4b), and quantification of these events permits the calculation of a genotype-specific pollen FGT (expressed as EGT events per seedling). In large-scale screens for EGT events in the germline, resistant seedlings (‘pollen EGT lines’) were found much more frequently when DSBR mutants served as pollen donors (Fig. 4c and Supplementary Table 6), indicating that defects in DSBR increase EGT in the male gametophyte. Remarkably, the increase in the EGT frequency was particularly strong (20-fold) in the *polq*_*ΔCDS*_*-3* and *polq*_*ΔHel*_*-4* mutants, demonstrating that the helicase domain of POLQ very strongly suppresses EGT, probably by preventing the formation of replication-associated DSBs independently of the polymerase domain of POLQ and the known mechanisms of TMEJ (as evidenced by the absence of a similar effect from the *polq* mutants defective in the polymerase domain).

Analysis of the segregation of kanamycin resistance in the progeny of pollen EGT lines revealed the presence of *nptII* as a single nuclear locus (Supplementary Table 7). Similar to the somatic EGT lines, pollen EGT lines occasionally displayed distorted segregation ratios of kanamycin resistance, due to stochastic *nptII* loss^57^ (Supplementary Table 5). Since NUPTs arising in the male gametophyte are directly transmitted to the progeny, our results indicate that alterations in genetic control change the speed of EGT over evolutionary timespans and potentially represent a substantial force in eukaryotic evolution.

## Discussion

In this study, we have shown that conserved eukaryotic DSBR pathways act as EGT suppressors. Our results suggest DSB availability as the key determinant of stable EGT and demonstrate that successful orgDNA integration into the nuclear genome is a bottleneck in EGT. Given that the occurrence of spontaneous DSBs is proportional to genome size^59^, our data also explain a previously reported general correlation between genome size and the number of NORGs in eukaryotic genomes^60,61^. We have also generated a large collection of plant lines that carry new insertions of orgDNA in the nuclear genome. These lines can now be employed to characterize the influence of DSBR on EGT with respect to position, size, stability, integration patterns and integration site preferences of newly incorporated orgDNA in the nucleus.

Our results reported here reveal two noteworthy aspects regarding the frequency of EGT. First, there is a striking time dependence of EGT frequencies in somatic cells, and second, impaired DSBR strongly affects EGT frequency in the germline. These two effects can lead to strong increases in EGT, compared with basal frequencies that had been recognized already as very high^62^. To our knowledge, the rates of integrative EGT measured in *lig4-2* somatic cells and in *polq*_*ΔCDS*_*-3* pollen are the highest reported to date. Moreover, given that our screen detects neither EGT from mitochondria nor EGT events from plastids that do not contain the *nptII* gene, our experimentally determined transfer frequencies are probably gross underestimates.

We conclude that the inactivation of POLQ (with a 20-fold increase in EGT), when combined with known interventions that compromise organelle integrity and/or promote orgDNA release^10^ (such as heat stress^63^), could result in extremely high germline EGT frequencies. The rate of EGT could become so high that the sheer number of orgDNA insertions would frequently interrupt nuclear genes and trigger genomic rearrangements. Consequently, orgDNA would act as a strong mutagen in the nucleus, thus making its suppression evolutionarily relevant. While DSBR evidently is important for reasons beyond EGT control, its role in the suppression of EGT should become significant when the mutagenic effects of orgDNA in the nucleus outweigh the evolutionary benefits of EGT.

The suppression of EGT by NHEJ and TMEJ relies on the reduction of DSB availability through fast repair^64^, which can be error prone. The potential instability-inducing role of orgDNA in the nucleus may explain why it is necessary to maximize the activity of both DSBR pathways: it is preferrable to suppress EGT through fast and possibly error-prone repair (occasionally leading to mutation), rather than allowing the frequent integration of orgDNA pieces that not only generate mutations upon insertion but may also drive genome instability thereafter. Since the NHEJ and MMEJ pathways are largely conserved in eukaryotes, our findings obtained here for tobacco are likely to apply to eukaryotes in general. Exceptions may exist in lineages where an individual DSBR pathway has been lost—for example, in the protozoan parasite *Toxoplasma*^29^, which seems to lack MMEJ.

In the course of this work, we also have generated a collection of DSBR mutants in tobacco. Previous studies of DNA repair in plants were centred on *Arabidopsis* or rice, and the results obtained with these two models were not always consistent. For example, POLQ has been reported as responsible for the integration of transfer DNA (T-DNA) in the *Arabidopsis* egg cell nucleus^40,65^, whereas results in rice *polq* mutants showed that T-DNA integration is not completely abolished in somatic cells^65^. In both species, *polq* mutants regenerate very poorly in tissue culture, and after considerable efforts, only a single fertile rice line derived from POLQ-independent T-DNA integration has been recovered^66^. By contrast, regeneration is unimpaired in our *polq*_*ΔPol*_ mutants and solely somewhat delayed in the helicase-defective *polq*_*ΔCDS*_*-3* and *polq*_*ΔHel*_*-4* mutants. Hence, our genetic material generated in tobacco will be useful for quantitative studies on the efficiency of T-DNA integration and other genetic manipulations such as the characterization of genome editing outcomes, as well as for genome biology (for example, by facilitating the study of the involvement of POLQ in replication stress tolerance).

In summary, we have discovered that eukaryotic DSBR pathways exert genetic control over EGT. They suppress the integration of orgDNA into the nuclear genome by limiting DSB availability in both somatic cells and the male germline. In addition, the very high EGT frequencies measured in our study suggest a strong mutagenic potential of orgDNA that poses a serious threat to nuclear genome stability and probably shaped the evolution of plant genomes.

## Methods

### Plant material and growth conditions

Tobacco plants (*N. tabacum* cv. Petit Havana) were grown in standard greenhouse conditions under a 16 h light / 8 h dark regime at ~300 μmol photons per m^2^ per s light intensity (day temperature, ~25 °C; night temperature, ~20 °C). Seed germination and cultivation in vitro were performed on agar-solidified Murashige and Skoog (MS) medium^67^ supplemented with antibiotics at appropriate concentrations (100–400 mg l^−1^ kanamycin or 75 mg l^−1^ hygromycin) and 3% sucrose (w/v). Plants were raised in sterile conditions on MS medium and grown in a controlled-environment chamber under a 16 h light / 8 h dark regime at 50 μmol photons per m^2^ per s (day temperature, ~25 °C; night temperature, ~20 °C).

### Tissue culture and plant regeneration

To regenerate plants from tobacco explants, leaves were cut into 25-mm^2^ pieces and regenerated on agar-solidified MS-based plant regeneration medium containing 3% (w/v) sucrose, 100 mg l^−1^ 1-naphtaleneacetic acid, 1 g l^−1^ 6-benzylaminopurine and the appropriate antibiotics (400 mg l^−1^ kanamycin or 15 mg l^−1^ hygromycin). Regenerated shoots were transferred to Magenta boxes containing MS medium with 3% sucrose (w/v) as the rooting medium.

### Identification of DSBR genes and guide RNA design for genome editing

Sequences of the *LIG4*, *POLQ*, *RPA1C*, *KU70* and *KU80* orthologues in *A. thaliana* (encoded by loci AT1G49250, AT4G32700, AT5G45400, AT1G16970 and AT1G48050, respectively) were used as queries in a search for orthologous sequences in draft genomes of *N. tabacum*^42,68^ using POTbase^69^ (https://chlorobox.mpimp-golm.mpg.de/potbase-application.html). Two tobacco homologues per *Arabidopsis* gene were identified and named *LIG4S* and *LIG4T*, *POLQS* and *POLQT*, and so on, according to sequence similarity with their orthologues in *N. sylvestris* (S) or *N. tomentosiformis* (T)^70^. The intron/exon structures of all loci were deduced from the associated transcriptomes. The gene models have been updated on the basis of the latest chromosome-level assembly of the *N. tabacum* genome^71^ (Supplementary Table 8). The coding sequences were used to generate pairs of guide RNAs (gRNAs) for gene editing (Supplementary Table 9). Target sequences were chosen that are fully conserved in both homologues of each gene.

### Isolation of nucleic acids and PCR

For genotyping reactions, genomic DNA was extracted from leaf tissue with the Extract-N-Amp kit (Sigma-Aldrich). We used 1 µl as the template for PCR in 25-µl reactions. For cloning and the initial PCR-based screening of mutations generated by Cas9, Phusion DNA polymerase (Thermo Fisher Scientific) was used. In all other PCR reactions, Dreamtaq DNA polymerase (Thermo Fisher Scientific) was used. PCR products were column-purified before sequencing (NucleoSpin Gel and PCR Clean-up, Macherey-Nagel).

### Construction of plant transformation vectors for tobacco genome editing

Plasmids were assembled for CRISPR–Cas-mediated genome editing of the tobacco *LIG4* loci (pEG021 and pEG022; Extended Data Fig. 2), the *POLQ* loci (Extended Data Fig. 3), the *RPA1C* loci (pEG025), the *KU70* loci (pEG028; Extended Data Fig. 6) and the *KU80* loci (pEG030; Extended Data Fig. 6). *POLQ* editing targeted the polymerase domain (pEG017 and pEG019), the helicase domain (pEG007) or both domains (pEG004). Briefly, an amplicon of plasmid pJF1046 (ref. ^72^) containing part of a gRNA scaffold and the U6 promoter and terminator was produced by PCR amplification introducing the sequences of gRNAs L and R, as well as BsaI sites at both ends, using specific primer pairs for each construct (Supplementary Tables 9 and 10). The PCR product was cloned into either pEG001 (ref. ^41^) or pEG1031 (ref. ^72^) through simultaneous cleavage of the BsaI sites and ligation^73^. pEG001 was used for the construction of plasmids pEG017, pEG019, pEG021, pEG022, pEG025, pEG028 and pEG030 to constitutively express *cas9* in plants; pJF1031 was used for the construction of pEG004 and pEG007 for egg-cell-specific *cas9* expression. The assembled plasmids are binary vectors that contain a T-DNA harbouring *cas9*, a hygromycin resistance marker for selection in planta and two cassettes for gRNA expression (gRNAs L and R).

### Generation of loss-of-function mutants in tobacco

Transplastomic Nt-RB98 plants with a wild-type nuclear background were super transformed with *Agrobacterium tumefaciens* strain GV2260 (ref. ^74^) containing the vector pEG004, pEG007, pEG017, pEG019, pEG021, pEG022, pEG025, pEG028 or pEG030 (Extended Data Fig. 1a) using the leaf disc infiltration method. Hygromycin-resistant plant lines were selected and maintained in medium containing hygromycin (15 mg l^−1^) and cefotaxime (250 mg l^−1^) to eliminate residual *Agrobacterium* cells. The screening for genome editing followed a strategy for the isolation of knockout alleles in the allotetraploid species tobacco without relying on visible phenotypes^41^. Extended Data Fig. 1b–d illustrates the PCR-based screen. Reactions 1–3 were used to characterize the mutations generated at the target sites L and R using specific primer pairs, whereas Reaction 4 was used for genotyping of *cas9* with oligonucleotides oEG095 and oEG298 (Supplementary Table 10).

For transformation experiments with constructs constitutively expressing *cas9* (pEG017, pEG019, pEG021, pEG022, pEG028 or pEG030), PCR and Sanger sequencing were used to identify Cas9 activity among the hygromycin-resistant primary transformants in T_0_ (‘transformed generation zero’). Reaction 1 was used to detect events of large deletions between the L and R sites. Next, Reaction 2 was used to identify further mutant alleles with small insertions or deletions at the L site. In all cases, a single additional regeneration step was performed to decrease the mosaicism in the tissue and fix the detected mutations. After the identification of regenerated shoots that carried similar mutations as the primary transformants, the regenerants were transferred to the greenhouse, and T_1_ seeds were obtained. Between 64 and 256 T_1_ seedlings per candidate stock were genotyped by performing Reactions 1, 2 and 4 (Extended Data Fig. 1c) to simultaneously assay for (and confirm) mutations at the L site, choose favourable allele combinations and segregate out the *cas9* transgene. Polymorphisms between the S and T loci allowed us to classify the amplified sequences as S or T homeoalleles after Sanger sequencing. When necessary, mutations at the gRNA R target site were analysed in T_1_ seedlings by Reaction 3. Linkage between mutations at sites L and R was determined by analysis of allele co-segregation patterns.

For transformations targeting the homologues of *KU70* (pEG028) and *KU80* (pEG030), phenotypes and their linkage to specific mutant alleles were analysed in T_1_ seedlings (representing a mix of *cas9*-containing and *cas9*-free plants). In all other cases, the *cas9* transgene was segregated out in the T_1_ generation.

Double homozygous lines were obtained in the T_1_ generation for *polq*_*ΔPol*_*-1* and *lig4-2* after transformation with pEG017 and pEG022, respectively, and in the T_2_ generation for *polq*_*ΔPol*_*-2* after transformation with pEG019. Seeds obtained from self-fertilization (selfing) of these lines were used for further experiments. The *lig4-1* knockout was isolated in the T_1_ generation after transformation with pEG021. This line was found to be homozygous for a large deletion in the S locus and heterozygous for mutant alleles in the T locus (one with a 61-nucleotide deletion in the L site, whereas in another one, the L site could not be amplified). A segregating *lig4-1* population in the T_2_ generation was used for the first somatic gene transfer experiment (Experiment 1). All other experiments with the *lig4-1* mutant were performed using a double homozygous derivative of *lig4-1* obtained from the T_2_ population, where the non-amplifiable allele had been segregated out.

For transformation experiments with constructs expressing *cas9* in the egg cell (pEG004 and pEG007), hygromycin-resistant plants were transferred to the greenhouse and self-fertilized to obtain T_1_ seeds. Cas9 activity was detected in T_1_ seedlings using Reaction 1. For pEG004, several lines with no remaining wild-type alleles were found in the T_1_ generation using Reaction 2, and a double homozygous, *cas9*-free mutant was obtained in the T_2_ generation (*polq*_*ΔPol*_*-3*). For pEG007, a single T_1_ plant (out of 192 tested) had a large in-frame deletion and no other mutations. After selfing, further frameshift mutations were found in the T_2_ progeny, and a double homozygous, *cas9*-free mutant was isolated in the T_3_ generation (*polq*_*ΔHel*_*-4*).

### Genotoxic stress tests

Surface-sterilized seeds from mutant lines and control tobacco plants were sown directly on medium containing 0, 0.5 or 2.5 mg l^−1^ bleomycin A5 hydrochloride (1,477.02 g mol^−1^; BLEOCIN Merck KGaA; Cat. No. 203408). Seedling phenotypes were assessed 17 days after sowing.

### Gene transfer experiments in tissue culture

In two large-scale experiments (Experiments 1 and 2), leaf material was harvested from tobacco plants grown for seven to eight weeks in sterile conditions. The leaves were cut with a sharp blade into 25-mm^2^ pieces and were transferred to round 100-mm-diameter selection plates containing 60 ml of agar-solidified regeneration medium supplemented with kanamycin (400 mg l^−1^), at a density of 12 leaf pieces per plate. The plates were sealed with plastic wrap and placed in a controlled-environment chamber for ~120 days (‘primary selection’) under a 16 h light / 8 h dark regime (day temperature, ~25 °C; night temperature, ~20 °C) at 25 μmol photons per m^2^ per s. After the first regenerants had appeared, the plates were surveyed regularly to retrieve regenerating material (calli or shoots) as it appeared (‘candidate lines’). The date of retrieval from the primary selection step was noted down for all candidate lines (‘harvest day’). To ensure that all EGT lines came from independent events, the ‘one event per leaf piece rule’ was applied: whenever a candidate line was retrieved from a plate, the leaf piece where it had come from was marked. At later time points, regenerating plant tissue that came from already marked leaf pieces was removed from the plates but not analysed. Regenerated tissue from the primary selection step was transferred to fresh selection medium (‘secondary selection’). Each candidate line was tested for regeneration capacity by exposing five explants to regeneration medium with 400 mg l^−1^ kanamycin (‘regeneration test’), and later transfer to fresh medium every 30 days. In addition, larger shoots (>4 mm) were transferred to rooting medium (MS plus 3% sucrose (w/v)) containing kanamycin (‘rooting test’). A line was scored as a true ‘EGT event’ if one of the following criteria was fulfilled during secondary selection: (1) primary shoots from the primary selection step rooted in boxes before day 90 (‘early rooting’), or (2) green shoots or abundant callus tissue regenerated from >50% of the explants taken from primary selection within 30 days. Calli and leaf material from all regenerating EGT lines were further propagated in an attempt to produce shoots for additional rooting attempts (for up to two more rounds of regeneration in fresh medium), or until a rooting plant was obtained. The lines that met criterion 2 were classified according to whether they had produced any rooting material (‘rooting after regeneration’) or not (‘positive by survival’). In Experiment 1, rooting was assessed for every EGT line at the latest 60 days after the final propagation. In Experiment 2, rooting of all EGT lines was assessed during a fixed period of 90 days starting from the initial recovery of the candidate line. A line was scored as an ‘escape’ if none of the above criteria for EGT lines were fulfilled (that is, escapes failed to regenerate green shoots or calli in regeneration tests, and usually bleached within 30 days in the rooting tests on kanamycin-containing medium).

To ensure direct comparability of somatic EGT measurements to those reported in previous studies, we adopted EGT events per hundred leaf pieces as a measure for the FGT in somatic cells. To transform leaf pieces into cell numbers, we applied the same conversion as in a previous report (one 25-mm^2^ leaf piece is equivalent to ~50,000 cells)^18^, which was based on cell counts in mature tobacco leaves^75^.

### Genetic archiving and seed tests

Up to a single rooted plant per somatic EGT line was transferred to the greenhouse to produce seeds (1) by using the EGT plant as the pollen donor in crosses to wild-type mothers to segregate out the Nt-RB98 plastid genome or (2) by selfing. Segregation of the kanamycin resistance trait in the progeny from the crosses was analysed after 18 days for up to 25 EGT lines per nuclear background, by sowing 160–260 seeds on medium containing 100 mg l^−1^ kanamycin.

### Gene transfer experiments in the male gametophyte

Plants derived from the Nt-RB98 background were grown in the greenhouse and used to manually pollinate emasculated wild-type flowers. In each independent cross, one to three individual plants received pollen from one to three donor plants of a single genotype. Seeds were sown on plates containing medium supplemented with kanamycin (100 mg l^−1^), at a density of ten seeds per ml of medium. After 18 days, kanamycin-resistant seedlings arising from EGT events in the male gametophyte were identified (‘pollen EGT lines’). Resistance was confirmed by rooting in fresh medium supplemented with 400 mg l^−1^ kanamycin. Segregation of the kanamycin resistance trait in the progeny of pollen EGT lines was analysed after 18 days of growth on medium containing 100 mg l^−1^ kanamycin.

### Statistics and modelling

Statistical models were assembled using R v.4.3.3 (https://www.R-project.org/). For modelling of the cumulative FGT over time and calculation of GTR, we retrospectively calculated the cumulative numbers of (1) candidates or (2) true EGT lines that had been recovered up to a certain sampling day. Because of the labour-intensive nature of the experiment, sampling did not occur in predefined intervals. Since the collection dates alone lead to unevenly distributed data points, we considered as data points the totals at the last harvest day of arbitrarily defined, non-overlapping windows of up to five days (Supplementary Table 2). Multiple linear regression models of cumulative FGT as a function of time and genotype were constructed with the maximum likelihood method in R (‘Models 1 and 2’). To account for the higher error associated with FGT measurements at earlier time points (when fewer EGT events had been recovered), the contribution of each time point was weighed by the square root of the total candidate lines obtained for that genotype up to that time point, using the ‘weights’ argument of the lm function.

For analysis of the effect of genotype on FGT in pollen, the minimal sample sizes per genotype for a sufficiently sensitive screen were determined through power analysis. Effect sizes were estimated by calculating Cohen’s *w*^76^. The desired sample sizes were defined as being sufficiently high to detect a change in GTR from 1:16,000 (the first reported frequency of EGT in tobacco pollen^20^) to 1:6,000 (*w* = 0.013) through pairwise comparisons with power 0.95 and *α* = 0.05, and subsequently Bonferroni-corrected for six comparisons. In this way, the minimal sample size was determined as 52,833 seeds per genotype. For analysis, counts of kanamycin-resistant and sensitive seedlings in the progeny of the crosses were modelled as proportions of binary outcomes using the binomial distribution (‘Model 3’).

All models were assembled using the glm and lm functions provided with the R Base Package (https://www.R-project.org/). Multiple *R*^2^ coefficients for the linear models were obtained from the lm output. Hypothesis testing of the differences between slopes for Models 1 and 2 was performed using the TestInteraction function of the phia package v.0.2-1. (https://CRAN.R-project.org/package=phia). For Model 3, hypothesis testing (the effects of each genotype relative to Nt-RB98) was performed using the cftest function of the multcomp package v.1.4-25 (https://CRAN.R-project.org/package=multcomp). We obtained 95% confidence intervals using the ciTools package v.0.6.1. (https://cran.r-project.org/package=ciTools). Statistical tests were performed using functions in the R Base Package (v.4.3.3), except the comparisons between slopes in Models 1 and 2, which were performed in R v.3.6.3, and the goodness-of-fit tests for the inheritance of the kanamycin resistance trait, which were calculated in Microsoft Excel.

### Availability of materials

Biological materials generated in the course of this work are available from the authors upon reasonable request.

### Reporting summary

Further information on research design is available in the Nature Portfolio Reporting Summary linked to this article.

## Supplementary information

Supplementary Information Supplementary Tables 1–10.

Reporting Summary

## Source data

Source Data Extended Data Fig. 5 Unprocessed agarose gels of the genotyping shown in Extended Data Fig. 5c.

## Data Availability

The data supporting the findings of this work are available within the paper, its extended data figures and its Supplementary Information. Sequences from *Arabidopsis* (AT1G49250.1, AT4G32700.2, AT5G45400.1, AT1G16970.1 and AT1G48050.1) are available through TAIR (https://www.arabidopsis.org/). Genomic sequences from *Nicotiana* (CM065996.1, CM065987.1, CM065979.1, CM066001.1, CM065985.1, CM065992.1 and CM065997.1) are available at GenBank (https://www.ncbi.nlm.nih.gov/genbank/). Accession numbers are also provided in the relevant sections of the paper. Source data are provided with this paper.

## Code availability

The R code used for assembling the statistical models is available via GitHub at https://github.com/egonzalezduran/modelsEGTdsbr.
