# Supplementary material for: Suppression of plastid-to-nucleus gene transfer by DNA double-strand break repair
Source: Nat Plants. 2025 May 16;11(6):1154–64. doi: 10.1038/s41477-025-02005-w (PMC12181080; doi:10.1038/s41477-025-02005-w)
Supplement: Supplementary file 1 — Supplementary Tables 1–10. [file 41477_2025_2005_MOESM1_ESM.pdf]

nature plants

Supplementary information

https://doi.org/10.1038/s41477-025-02005-w

Suppression of plastid-to-nucleus gene transfer by DNA double-strand break repair

In the format provided by the authors and unedited

Table of contents:

Supplementary Tables 1-10

Supplementary Table 1. Linear models and parameter values for calculation of GTR in somatic cells.

										Hypothesis testing (*df* =1)			
Model	Multiple R^2	Genotype	Parameter	Value	Standard Error	CI95 lower bound	CI95 upper bound	Wald *z*-statistic	*P*-value	Slope comparison	Fold change	X^2	*P*-value
Model 1 (Experiment 1)	0.997	Nt-RB98	GTR	8.49 x 10^{-9}	8.59 x 10^{-10}	6.81 x 10^{-9}	1.02 x 10^{-8}	9.88	8.59 x 10^{-11}	*polq*$_{\Delta Pol}$*-1* vs Nt-RB98	1.6	18.59	1.6 x 10^{-5}
			Intercept	-3.81 x 10^{-7}	7.92 x 10^{-8}	-5.36 x 10^{-7}	-2.25 x 10^{-7}	-4.81	4.35 x 10^{-5}				
		polq$_{\Delta Pol}$*-1*	GTR	1.34 x 10^{-8}	7.48 x 10^{-10}	1.19 x 10^{-8}	1.49 x 10^{-8}	17.93	3.11 x 10^{-17}	*lig4-1* vs Nt-RB98	2.9	194.66	3.1 x 10^{-44}
			Intercept	-6.28 x 10^{-7}	7.25 x 10^{-8}	-7.70 x 10^{-7}	-4.86 x 10^{-7}	-8.66	1.55 x 10^{-9}				
		lig4-1	GTR	2.43 x 10^{-8}	7.43 x 10^{-10}	2.29 x 10^{-8}	2.58 x 10^{-8}	32.77	1.83 x 10^{-24}	*lig4-1* vs *polq*$_{\Delta Pol}$*-1*	1.8	107.67	3.2 x 10^{-25}
			Intercept	-9.20 x 10^{-7}	7.24 x 10^{-8}	-1.06 x 10^{-6}	-7.78 x 10^{-7}	-12.69	2.29 x 10^{-13}				
Model 2 (Experiment 2)	0.994	Nt-RB98	GTR	5.16 x 10^{-9}	1.17 x 10^{-9}	2.86 x 10^{-9}	7.47 x 10^{-9}	4.40	1.64 x 10^{-4}	*polq*$_{\Delta Pol}$*-2* vs Nt-RB98	3.2	45.97	2.4 x 10^{-11}
			Intercept	-7.35 x 10^{-8}	1.11 x 10^{-7}	-2.91 x 10^{-7}	1.44 x 10^{-7}	-0.66	0.51417				
		polq$_{\Delta Pol}$*-2*	GTR	1.69 x 10^{-8}	1.27 x 10^{-9}	1.44 x 10^{-8}	1.94 x 10^{-8}	13.31	4.06 x 10^{-13}	*lig4-2* vs Nt-RB98	5.5	165.38	7.7 x 10^{-38}
			Intercept	-4.49 x 10^{-7}	1.18 x 10^{-7}	-6.80 x 10^{-7}	-2.17 x 10^{-7}	-3.79	7.99 x 10^{-4}				
		lig4-2	GTR	2.85 x 10^{-8}	1.38 x 10^{-9}	2.57 x 10^{-8}	3.12 x 10^{-8}	20.63	1.21 x 10^{-17}	*lig4-2* vs *polq*$_{\Delta Pol}$*-2*	1.7	38.18	6.5 x 10^{-10}
			Intercept	-1.05 x 10^{-6}	1.29 x 10^{-7}	-1.30 x 10^{-6}	-7.96 x 10^{-7}	-8.12	1.33 x 10^{-8}				

The gene transfer rate (GTR) corresponds to the slope of the linear model and is expressed in units of [EGT events cell^{-1} day^{-1}]. Intercept units are [EGT events cell^{-1}]. *df*: degrees of freedom. Hypotheses pertaining to a single model were tested simultaneously through calculation of X^2 (chi-square) statistics (see Methods); *P*-values are corrected for multiple comparisons using the Holm method.

Supplementary Table 2. Cumulative counts of EGT events and putative escapes during primary selection in EGT experiments in tissue culture.

Experiment 1														
Nt-RB98						*61*								
(NIp*:* 4272)						*62*		*82*			*105*	*118*		
			44		*56*	*63*		*84*		*99*	*106*	*119*		
	Day of harvest	***28***	***47***	***51***	***57***	***65***	***70***	***85***	***91***	***100***	***107***	***120***		
	Candidate lines	2	38	43	67	176	192	317	338	393	571	766		
	EGT events	1	10	10	14	40	41	74	81	94	119	137		
	Escapes	1	28	33	53	136	151	243	257	299	452	629		
polq$_{\Delta Pol}$*-1*				*53*						*95*	*102*			
(NIp*:* 5112)			*49*	*55*	*61*	*67*		*84*		*96*	*103*	*110*	*117*	
	Day of harvest	***34***	***50***	***56***	***64***	***70***	***77***	***85***	***91***	***97***	***104***	***111***	***118***	***124***
	Candidate lines	12	41	103	125	205	219	278	294	336	454	537	651	669
	EGT events	3	17	41	49	89	93	124	136	154	190	233	257	264
	Escapes	9	24	62	76	116	126	154	158	182	264	304	394	405
lig4-1			*54*											
(NIp*:* 4544)			*55*	*62*				*91*		*11*	*119*			
			57	*63*	*71*		*85*	*92*	*97*	*113*	*120*			
	Day of harvest	***35***	***58***	***64***	***72***	***80***	***86***	***94***	***98***	***114***	***121***	***124***		
	Candidate lines	24	131	228	276	349	404	454	521	595	706	722		
	EGT events	16	82	139	172	232	271	328	366	404	461	468		
	Escapes	8	49	89	104	117	133	147	155	191	245	254		
Experiment 2														
Nt-RB98			*49*		*63*									
(NIp: 2345)	Day of harvest	***42***	***50***	***58***	***64***	***70***	***77***	***84***	***89***	***98***	***103***	***125***	***128***	
	Candidate lines	19	55	104	150	179	220	229	251	295	339	422	489	
	EGT events	8	14	27	31	35	41	44	46	52	57	62	69	
	Escapes	11	41	77	119	144	179	185	205	243	282	360	420	
polq$_{\Delta Pol}$*-2*			*48*						*94*		*124*			
(NIp: 2248)	Day of harvest	***38***	***49***	***56***	***68***	***76***	***81***	***88***	***95***	***119***	***125***			
	Candidate lines	27	59	96	145	191	246	279	361	461	511			
	EGT events	11	26	48	72	93	113	129	150	166	178			
	Escapes	16	33	48	73	98	133	150	211	295	333			
lig4-2				*62*	*68*	*75*	*83*			*117*				
(NIp: 2292)	Day of harvest	***41***	***49***	***63***	***69***	***76***	***84***	***88***	***96***	***118***	***124***			
	Candidate lines	14	48	111	154	210	267	311	352	445	481			
	EGT events	7	28	66	91	127	163	189	213	257	271			
	Escapes	7	20	45	63	83	104	122	139	188	210			

NIp*:* Number of leaf pieces. Numbers in italics correspond to a day (relative to the start of the experiment), on which candidate lines were harvested. To reduce the influence of highly clustered time points in the confidence intervals obtained from modelling (Supplementary Table 1), the points were arbitrarily combined in groups spanning a maximum period of 5 days, without overlap between groups. Only the final harvest time point of each group (italics and bold) was considered for analysis.

Supplementary Table 3. Number of lines and seed stocks obtained from somatic EGT experiments.

Lines	Experiment 1			Experiment 2			Total
	Nt-RB98	*polq$_{\Delta Pol}$-1*	*lig4-1*	Nt-RB98	*polq$_{\Delta Pol}$-2*	*lig4-2*	
Total candidate lines	766	669	722	489	511	481	3,638
EGT events	137	264	468	69	178	271	1,387
Positive by survival	9	44	190	14	32	76	365
Early rooting	48	92	98	31	58	86	413
Rooting after regeneration	80	128	180	24	88	109	609
Escapes	629	405	254	420	333	210	2,251
Rooted lines	128	220	278	55	146	195	1,022
Transferred to the greenhouse	125	207	226	54	145	188	945
With seeds produced	109	176	224	51	139	172	871
From crossing* and selfing	79	114	147	45	119	156	660
Crossing* only	9	26	32	6	20	14	107
Selfing only	21	36	45	0	0	2	104
No seeds recovered	16	31	2	3	6	16	74

*Crosses in which the EGT line is the pollen donor to an emasculated wild-type maternal recipient (WT x EGT line).

Supplementary Table 4. Segregation of the kanamycin resistance trait in the progeny (F_1) of wild-type maternal recipients cross-fertilized with pollen of plants derived from somatic EGT events.

Background of EGT line	EGT line	Resistant	Sensitive	% Resistance	kr: ks ratio (empirical)	χ^2 (df = 1)	P-value	
Nt-RB98	*13*	114	106	52%	1.1 : 1	0.29	0.590	
(n=25)	*73*	105	115	48%	1 : 1.1	0.45	0.500	
	85	98	122	45%	1 : 1.2	2.62	0.106	
	88	71	150	32%	1 : 2.1	28.24	1×10^{-7}	****
	127	104	116	47%	1 : 1.1	0.65	0.418	
	158	6	214	3%	1 : 35.7	196.65	1×10^{-44}	****
	203	104	112	48%	1 : 1.1	0.30	0.586	
	232	48	173	22%	1 : 3.6	70.70	1×10^{-17}	****
	253	108	110	50%	1 : 1	0.02	0.892	
	269	102	115	47%	1 : 1.1	0.78	0.378	
	283	101	114	47%	1 : 1.1	0.79	0.375	
	292	118	102	54%	1.2 : 1	1.16	0.281	
	294	104	116	47%	1 : 1.1	0.65	0.418	
	295	106	113	48%	1 : 1.1	0.22	0.636	
	302	68	151	31%	1 : 2.2	31.46	2×10^{-8}	****
	311	0	220	0%	-	>216.02	$< 7 \times 10^{-49}$	****
	364	112	121	48%	1 : 1.1	0.35	0.555	
	397	45	174	21%	1 : 3.9	75.99	$3. \times 10^{-18}$	****
	471	94	120	44%	1 : 1.3	3.16	0.076	
	507	117	103	53%	1.1 : 1	0.89	0.345	
	567	92	126	42%	1 : 1.4	5.30	0.021	*
	632	115	105	52%	1.1 : 1	0.45	0.500	
	645	113	105	52%	1.1 : 1	0.29	0.588	
	657	1	219	0.5%	1 : 219	216.02	$7 \times .10^{-49}$	****
	769	100	98	51%	1 : 1	0.02	0.887	
						Lines with the expected segregation ratio (1:1)	17	68%
						Less resistance than expected	7	28%
						Resistance lost	1	4%
Background of EGT line	EGT line	Resistant	Sensitive	% Resistance	kr: ks ratio (empirical)	χ^2 (df = 1)	P-value	
polq$_{\Delta Pol}$*-1*	*16*	114	103	53%	1.1 : 1	0.56	0.455	
(n=25)	*57*	109	103	51%	1.1 : 1	0.17	0.680	
	72	85	86	50%	1 : 1	0.01	0.939	
	97	104	110	49%	1 : 1.1	0.17	0.682	
	110	107	109	50%	1 : 1	0.02	0.892	
	126	89	129	41%	1 : 1.4	7.34	0.007	**
	140	56	114	33%	1 : 2	19.8	9×10^{-6}	****
	177	102	108	49%	1 : 1.1	0.17	0.679	
	183	104	110	49%	1 : 1.1	0.17	0.682	
	191	102	106	49%	1 : 1	0.08	0.782	
	228	70	86	45%	1 : 1.2	1.641	0.200	
	289	5	205	2%	1 : 41	182.3	2×10^{-41}	****
	291	0	220	0%	-	>216.02	$< 7 \times 10^{-49}$	****
	334	91	119	43%	1: 1.3	3.733	0.0533	
	355	106	132	45%	1 : 1.2	2.840	0.0919	
	357	111	113	50%	1 : 1	0.018	0.894	
	400	90	134	40%	1 : 1.5	8.643	0.00328	**
	476	57	156	27%	1 : 2.7	46.014	1.2×10^{-11}	****
	483	14	206	6%	1 : 14.7	167.6	3×10^{-38}	****
	496	55	170	24%	1 : 3.1	58.78	8.7×10^{-6}	****
	506	113	107	51%	1.1: 1	0.309	0.686	
	522	105	113	48%	1 : 1.1	0.31	0.578	
	538	116	124	48%	1 : 1.1	0.267	0.606	
	632	89	121	42%	1 : 1.4	4.876	0.0272	*
	686	99	111	47%	1 : 1.1	0.686	0.408	
						Lines with the expected segregation ratio (1:1)	16	64%
						Less resistance than expected	8	32%
						Resistance lost	1	4%
Background of EGT line	EGT line	Resistant	Sensitive	% Resistance	kr: ks ratio (empirical)	χ^2 (df = 1)	P-value	
polq$_{\Delta Pol}$*-2*	*9*	62	154	29%	1 : 2.5	39.185	3.9×10^{-10}	****
(n=24)	*23*	73	119	38%	1 : 1.6	11.021	9.0×10^{-4}	****
	72	1	217	0%	1 : 217	214.018	1.8×10^{-48}	****
	61	0	208	0%	-	>204.019	$< 2.8 \times 10^{-46}$	****
	77	79	131	38%	1 : 1.7	12.876	3.3×10^{-4}	****
	82	97	121	44%	1 : 1.2	2.642	0.104	
	127	0	226	0%	-	>222.018	$< 3.3 \times 10^{-50}$	****
	149	3	195	2%	1 : 65	186.182	2.2×10^{-42}	****
	150	6	181	3%	1 : 30.2	163.770	1.7×10^{-37}	****
	156	0	222	0%	-	218.018	$< 2.4 \times 10^{-49}$	****
	183	115	103	53%	1.1 : 1	0.661	0.416	

	202	42	164	20%	1 : 3.9	72.252	1.9 x 10^{-17}	****
	209	97	111	47%	1 : 1.1	0.942	0.332	
	214	87	104	46%	1 : 1.2	1.513	0.219	
	221	78	113	41%	1 : 1.4	6.414	0.011	*
	259	0	194	0%	-	>190.021	< 3.4 x 10^{-43}	****
	266	93	128	42%	1 : 1.4	5.543	0.019	*
	303	110	106	51%	1 : 1	0.074	0.785	
	328	119	91	57%	1.3 : 1	3.733	0.053	
	352	102	95	52%	1.1 : 1	0.249	0.618	
	396	8	209	4%	1 : 26.1	186.180	2.2 x 10^{-42}	****
	464	105	103	50%	1 : 1	0.019	0.890	
	493	114	103	53%	1.1 : 1	0.558	0.455	
	511	67	145	32%	1 : 2.2	28.698	8.5 x 10^{-8}	****
						Lines with the expected segregation ratio (1:1)	9	38%
						Less resistance than expected	11	46%
						Resistance lost	4	17%

Background of EGT line	EGT line	Resistant	Sensitive	% Resistance	kr: ks ratio (empirical)	χ^2 (*df* = 1)	*P*-value	
lig4-1 (n=25)	*2*	74	94	42%	1 : 1.3	2.809	0.094	
	13	87	74	52%	1.1 : 1	0.304	0.581	
	18	82	93	47%	1 : 1.1	0.691	0.406	
	33	81	74	52%	1.1 : 1	0.316	0.574	
	37	50	71	41%	1 : 1.4	3.645	0.056	
	43	74	60	51%	1.2 : 1	2.056	0.152	
	52	51	64	44%	1 : 1.3	1.470	0.225	
	61	64	65	50%	1 : 1	0.008	0.930	
	98	90	76	54%	1.2 : 1	1.181	0.277	
	107	72	70	51%	1 : 1	0.028	0.867	
	158	78	88	47%	1 : 1.1	0.602	0.438	
	172	26	142	15%	1 : 5.5	80.095	3.5 x 10^{-19}	****
	192	86	82	51%	1 : 1	0.095	0.758	
	234	86	80	52%	1.1 : 1	0.217	0.641	
	241	78	74	51%	1.1 : 1	0.105	0.746	
	244	95	73	57%	1.3 : 1	2.881	0.090	
	268	73	59	55%	1.2 : 1	1.485	0.223	
	304	65	62	51%	1 : 1	0.071	0.790	
	334	70	69	50%	1 : 1	0.007	0.932	
	353	26	97	21%	1 : 3.7	40.984	1.5 x 10^{-10}	****
	376	75	71	51%	1.1 : 1	0.110	0.741	
	385	102	106	49%	1 : 1	0.077	0.782	
	421	72	83	46%	1 : 1.2	0.781	0.377	
	531	95	97	49%	1 : 1	0.021	0.885	
	682	92	101	48%	1 : 1.1	0.420	0.517	
						Lines with the expected segregation ratio (1:1)	23	92%
						Less resistance than expected	2	8%
						Resistance lost	0	0%

Background of EGT line	EGT line	Resistant	Sensitive	% Resistance	kr: ks ratio (empirical)	χ^2 (*df* = 1)	*P*-value	
lig4-2 (n=21)	*41*	48	171	22%	1 : 3.6	69.082	9.4 x 10^{-17}	****
	55	107	104	51%	1 : 1	0.043	0.836	
	62	89	127	41%	1 : 1.4	6.685	9.7 x 10^{-3}	**
	67	102	94	52%	1.1 : 1	0.327	0.568	
	81	107	109	50%	1 : 1	0.019	0.892	
	87	96	123	44%	1 : 1.3	3.329	0.0681	
	89	101	115	47%	1 : 1.1	0.907	0.341	
	136	123	96	56%	1.3 : 1	3.329	0.0681	
	141	1	192	1%	1 : 192	189.021	5.2 x 10^{-43}	****
	197	112	112	50%	1 : 1	0	1	
	207	98	110	47%	1 : 1.1	0.692	0.405	
	219	96	116	45%	1 : 1.2	1.887	0.170	
	237	115	100	53%	1.2 : 1	1.047	0.306	
	246	105	113	48%	1 : 1.1	0.294	0.588	
	257	106	98	52%	1.1 : 1	0.314	0.575	
	322	97	103	49%	1 : 1.1	0.180	0.671	
	376	110	78	59%	1.4 : 1	5.447	0.0196	*
	431	52	161	24%	1 : 3.1	55.779	8.1 x 10^{-14}	****
	446	100	105	49%	1 : 1.1	0.122	0.727	
	455	90	101	47%	1 : 1.1	0.634	0.426	
	466	100	102	50%	1 : 1	0.020	0.888	
						Lines with the expected segregation ratio (1:1)	16	76%
						Higher resistance than expected	1	5%
						Less resistance than expected	4	19%
						Resistance lost	0	0%

Seedlings were selected on media containing 100 mg/L kanamycin. Numbers in italics correspond to the name of the pollen donor, assigned when the EGT line was recovered as a candidate line in the primary selection. Deviations from the expected segregation ratio (1:1) were analyzed through one-sided χ^2 (chi-square) tests. P-values are calculated without correction. *df*: degrees of freedom. kr: kanamycin resistant, ks: kanamycin sensitive. *, $P < 0.05$; **, $P < 0.01$; ***, $P < 0.001$; ****, $P < 0.0001$.

Supplementary Table 5. Presence of the *nptII* amplicon in the progeny of EGT lines displaying distorted segregation of the kanamycin resistance trait.

Experimental origin of EGT line	Segregation ratio	Progenitor EGT line	kr : ks ratio (empirical)	Genotyping (*nptII*+ samples/samples analyzed) From kr seedlings	Genotyping (*nptII*+ samples/samples analyzed) From ks seedlings	Expected proportion of *nptII*+ among genotyped ks seedlings (assuming gene silencing)	χ^2 (df = 1)	*P*-value	
Somatic EGT	Expected (1 : 1)	Nt-RB98#13	1.1 : 1	16/16	0/16				
		$polq_{\Delta Pol}$-1#16	1.1 : 1	16/16	0/16				
	Distorted	Nt-RB98#158	1 : 35.7	1/1	2/31	15/31	6.524	0.0106	*
		Nt-RB98#397	1 : 3.9	1/1	0/31	11/31	7.623	0.0057	**
		$polq_{\Delta Pol}$-1#289	1 : 41	1/1	0/31	15/31	10.562	0.0011	**
		$polq_{\Delta Pol}$-1#483	1 : 14.1	1/1	0/31	14/31	9.843	0.0017	**
		$polq_{\Delta Pol}$-2#9	1 : 2.5	1/1	0/31	9/31	6.084	0.0136	*
		$polq_{\Delta Pol}$-2#202	1 : 3.9	1/1	11/31	12/31	6×10^{-31}	1	
		lig4-1#172	1 : 5.5	1/1	0/31	13/31	9.114	0.0025	**
		lig4-1#353	1 : 3.7	1/1	0/31	11/31	7.623	0.0057	**
		lig4-2#41	1 : 3.6	1/1	0/31	11/31	7.623	0.0057	**
		lig4-2#431	1 : 3.1	1/1	0/31	10/31	6.860	0.0088	**
EGT in male gametophyte	Expected (3 : 1)	(WT x Nt-RB98)#1	3.7 : 1	16/16	0/16				
	Distorted	(WT x Nt-RB98)#2	1.8 : 1	1/1	7/31	9/31	0.027	0.8686	
		(WT x Nt-RB98)#3	1.4 : 1	1/1	0/31	12/31	8.374	0.0038	**
		(WT x *lig4-2)#3*	1.3 : 1	1/1	0/31	13/31	9.114	0.0025	**
		(WT x *lig4-2)#5*	1.3 : 1	1/1	0/31	13/31	9.114	0.0025	**
		(WT x *lig4-2)#12*	Resistance lost	-	0/32	16/32	11.271	0.0008	***
						Lines with distorted segregation ratios analyzed		15	
						Lines displaying evidence against gene silencing as sole explanation for distorted segregation (P<0.05)		13	

WT: wild type. kr: kanamycin resistant, ks: kanamycin sensitive. *nptII* was amplified by PCR using oligos oEG381 and oEG382. For the expected segregation ratios of kanamycin resistance in self-fertilized plants (3:1) and crosses (1:1), it is assumed that the *nptII* insertion is neither gametophytically lethal nor recessively lethal to the embryo. The hypothesis stating that the distorted ratios are the result of the loss of the *nptII* gene was tested through contingency tables and one-sided χ^2 (chi-square) tests. The null hypothesis is that the ks seedlings in excess (responsible for the distortion) have retained and silenced the *nptII* gene, in which case the allele should be found at predictable proportions among ks seedlings. Rejection of the null hypothesis ($P < 0.05$) is considered as evidence for genetic instability of the *nptII* locus. Calculations of expected proportions of nptII+/total among ks seedlings are based on the counts of kr and ks seedlings in Supplementary Table 4 (somatic EGT lines) and Supplementary Table 7 (pollen EGT lines). The expected representation of *nptII*+ among ks seedlings was calculated using the following formula: [(Number of seedlings screened for kanamycin resistance x Expected proportion of resistant seedlings – Number of resistant seedlings)/ Number of sensitive seedlings]. The expected proportions are presented as fractions, where the numerator is the expected number of *nptII*+ ks seedlings (rounded to the nearest integer) for χ^2 calculation. *df*: degrees of freedom. *P*-values are calculated without corrections. *, $P < 0.05$; **, $P < 0.01$; ***, $P < 0.001$; ****, $P < 0.0001$.

Supplementary Table 6. Counts of EGT events that likely occurred during pollen development in DSBR mutants.

						Comparison to Nt-RB98		
Cross	Replicate	Kanamycin-resistant seedlings	Total seedlings screened	Ratio resistant to sensitive	FGT [CI95] (EGT events/seedling)	Fold-change	Wald z-statistic	P-value
WT x Nt-RB98	First	1	75,200					
	Second	2	79,200					
	Total	3	154,400	1 in 51,466	1.9×10^{-5} [6.3×10^{-6} — 6.0×10^{-5}]	-	-	-
WT x *lig4-1*		12	89,400	1 in 7,450	1.3×10^{-4} [7.6×10^{-5} — 2.4×10^{-4}]	6.9	2.994	0.00825**
WT x *lig4-2*		12	72,400	1 in 6,033	1.7×10^{-4} [9.4×10^{-5} — 2.9×10^{-4}]	8.5	3.321	0.00359**
WT x $polq_{\Delta Pol}$-1	First	4	54,600					
	Second	5	40,000					
	Total	9	94,600	1 in 10,511	9.5×10^{-5} [5.0×10^{-5} — 1.8×10^{-4}]	4.9	2.383	0.0219*
WT x $polq_{\Delta Pol}$-2	First	5	60,800					
	Second	6	47,200					
	Total	11	108,000	1 in 9,818	1×10^{-4} [6×10^{-5} — 1.8×10^{-4}]	5.2	2.544	0.0219*
WT x $polq_{\Delta CDS}$-3		25	65,600	1 in 2,624	3.8×10^{-4} [2.6×10^{-4} — 5.6×10^{-4}]	19.6	4.871	6.7×10^{-6} ****
WT x $polq_{\Delta Hel}$-4		22	62,800	1 in 2,855	3.5×10^{-4} [2.3×10^{-4} — 5.3×10^{-4}]	18.0	4.699	1.3×10^{-5} ****

WT: wild type. Seedlings were selected on media containing 100 mg/L kanamycin. A total of 83 (out of 95) putative EGT lines were transferred to fresh medium containing 400 mg/L kanamycin. 82 out of 83 transferred lines displayed robust kanamycin resistance. The remaining sensitive line (from the WT x *lig4-2* cross) was excluded from this table and the calculation of FGT in Figure 4c. CI95 and *P*-values from comparisons are obtained from analysis of Model 3 (see Methods). *P*-values are obtained from two-sided Wald *z*-tests and are corrected for multiple comparisons using the Holm method. *, $P < 0.05$; **, $P < 0.01$; ***, $P < 0.001$; ****, $P < 0.0001$

Supplementary Table 7. Segregation of the kanamycin resistance trait in the progeny of self-fertilized plants derived from EGT events in the male gametophyte.

Cross of origin	Pollen EGT line	Progeny from self-fertilization of EGT line: Resistant	Sensitive	% Resistance	kr : ks ratio (empirical)	χ^2	*P*-value	
WT x Nt-RB98 (n=3)	*1*	164	44	79%	3.7 : 1	1.641	0.2002	
	2	133	74	64%	1.8 : 1	12.755	4×10^{-4}	***
	3	60	43	58%	1.4 : 1	15.408	8×10^{-5}	****
WT x *lig4-1* (n=12)	*1*	163	45	78%	3.6 : 1	1.256	0.2623	
	2	155	49	76%	3.2 : 1	0.105	0.7464	
	3	166	40	81%	4.2 : 1	3.424	0.0643	
	4	162	49	77%	3.3 : 1	0.355	0.5510	
	5	154	54	74%	2.9 : 1	0.103	0.7488	
	6	157	46	77%	3.4 : 1	0.593	0.4413	
	7	149	46	76%	3.2 : 1	0.207	0.6493	
	8	144	60	71%	2.4 : 1	2.118	0.1456	
	9	142	62	70%	2.3 : 1	3.163	0.0753	
	10	159	48	77%	3.3 : 1	0.362	0.5472	
	11	154	47	77%	3.3 : 1	0.280	0.5965	
	12	156	45	78%	3.5 : 1	0.731	0.3924	
WT x *lig4-2* (n=11)	*1*	145	46	76%	3.2 : 1	0.086	0.7700	
	2	138	56	71%	2.5 : 1	1.546	0.2137	
	3	91	68	57%	1.3 : 1	26.769	2×10^{-7}	****
	4	143	51	74%	2.8 : 1	0.172	0.6785	
	5	113	84	57%	1.3 : 1	32.692	1×10^{-8}	****
	6	148	42	78%	3.5 : 1	0.849	0.3568	
	7	156	46	77%	3.4 : 1	0.535	0.4647	
	8	133	48	73%	2.8 : 1	0.223	0.6369	
	9	102	30	77%	3.4 : 1	0.364	0.5465	
	11	148	65	69%	2.3 : 1	3.457	0.0630	
	12	0	190	0%	-	565.028	$< 6 \times 10^{-124}$	****
						Lines with the expected segregation ratio (3:1)	21	
						Lines with less resistance than expected	4	
						Resistance lost	1	

WT: wild type. kr: kanamycin resistant, ks: kanamycin sensitive. Seeds derived from the self-fertilization of pollen EGT lines (Supplementary Table 6) were selected on media containing 100 mg/L kanamycin. Numbers in italics correspond to the name of the of the pollen EGT line, assigned when they were found to be kanamycin-resistant in the pollen EGT screens. This table includes data of all pollen EGT lines for which seeds are currently available (n=26): plants derived from EGT events in WT x *polq* crosses, as well as line WT x *lig4-2#10*, are currently setting seed and could not be included in this analysis. The single plant with the resistance lost in the progeny (WT x *lig4-2#12*) had itself shown robust resistance to 400 mg/L kanamycin, and is included as a true EGT event in Figure 4c and Supplementary Table 6. All lines showed high germination rates (95%>) except for WT x Nt-RB98#3 (50%). Deviations from the expected 3:1 ratio were determined through one-sided χ^2 (chi-square) tests. *P*-values are calculated without correction for multiple comparisons. *, $P < 0.05$; **, $P < 0.01$; ***, $P < 0.001$; ****, $P < 0.0001$.

Supplementary Table 8. Gene model sequences of tobacco DSBR genes .

		Coordinates	
Homolog	Chromosome (GenBank accession)	Genomic (orientation)	Coding sequence
LIG4S	Chromosome 18 (CM065996.1)	46831386..46881642 (forward)	46831386..46831760, 46833732..46833823, 46847611..46847696, 46847781..46847917, 46851040..46851114, 46851261..46851352, 46852266..46852337, 46853063..46853142, 46853300..46853337, 46853575..46853728, 46857593..46857668, 46860510..46860634, 46860716..46860750, 46860846..46860904, 46862540..46862623, 46863307..46863402, 46863478..46863526, 46863790..46863877, 46864000..46864203, 46864551..46864676, 46865232..46865331, 46865437..46865542, 46865682..46865812, 46865928..46866092, 46879150..46879305, 46880946..46881642
LIG4T	Chromosome 9 (CM065987.1)	37341524..37302512 (reverse)	37341524..37341150, 37338929..37338838, 37333575..37333490, 37333396..37333260, 37330215..37330141, 37330002..37329911, 37328975..37328904, 37328216..37328137, 37327978..37327941, 37327708..37327551, 37319339..37319264, 37316292..37316168, 37316086..37316052, 37315956..37315898, 37310749..37310666, 37310265..37310170, 37310094..37310046, 37309783..37309696, 37309573..37309367, 37309016..37308891, 37308357..37308258, 37308152..37308047, 37307906..37307776, 37307660..37307496, 37304687..37304532, 37303208..37302512
POLQS	Chromosome 1 (CM065979.1)	75245020..75279711 (forward)	75245020..75245055, 75247586..75248013 ,75248199..75248367, 75248451..75249329, 75253230..75253294, 75255993..75256098, 75256195..75256395, 75256588..75256788, 75264948..75265109, 75265200..75265439, 75265611..75265823, 75266121..75266471, 75266631..75266783, 75267813..75268028, 75270020..75270238, 75270315..75270432, 75271006..75272144, 75272448..75272645, 75273758..75274017, 75275528..75275717, 75275815..75275925, 75276003..75276355, 75278235..75278343, 75278454..75278591, 75279309..75279405, 75279641..75279711
POLQT	Chromosome 23 (CM066001.1)	87223943..87195555 (reverse)	87223943..87223908, 87222623..87222196, 87222078..87221892, 87221816..87220938, 87217361..87217297, 87213990..87213885, 87213787..87213587, 87213394..87213194, 87207587..87207426, 87207334..87207095, 87206916..87206704, 87206405..87206053, 87205891..87205741, 87204703..87204488, 87203788..87203570, 87203493..87203376, 87202811..87201673, 87201457..87201260, 87200183..87199924, 87199578..87199389, 87199290..87199180, 87199100..87198748, 87197062..87196954, 87196837..87196700, 87195974..87195878, 87195625..87195555
KU70S	Chromosome 7 (CM065985.1)	3056870..3073391 (forward)	3056870..3056940, 3059230..3059335, 3061422..3061536, 3062959..3063064, 3063177..3063294, 3063488..3063571, 3063649..3063707, 3065684..3065817, 3065909..3065952, 3067425..3067501, 3068608..3068755, 3069273..3069334, 3070580..3070640, 3071456..3071548, 3071649..3071766, 3072560..3072662, 3072793..3072964, 3073278..3073391
KU70T	Chromosome 14 (CM065992.1)	1505191..1485508 (reverse)	1505191..1505120, 1501967..1501863, 1497927..1497813, 1496302..1496197, 1496083..1495966, 1495773..1495690, 1495613..1495555 ,1493542..1493409, 1493317..1493274, 1490851..1490775, 1489709..1489562, 1489031..1488970, 1488369..1488309, 1487486..1487394, 1487291..1487174, 1486338..1486236, 1486107..1485936, 1485621..1485508
KU80S	Chromosome 7 (CM065985.1)	159353821..159364099 (forward)	159353821..159353877, 159353987..159354038, 159354620..159354751, 159360341..159360744, 159361519..159361805, 159361927..159362122, 159362223..159362429, 159363048..159363215, 159363324..159363653, 159363754..159363884, 159363979..159364099
KU80T	Chromosome 19 (CM065997.1)	98307792..98302542 (reverse)	98307792..98307736, 98307628..98307577, 98307001..98306870, 98306375..98305972, 98305116..98304830, 98304708..98304513, 98304412..98304206, 98303585..98303418, 98303313..98302984, 98302883..98302753, 98302662..98302542
RPA1CS	Chromosome 1 (CM065979.1)	116598204..116601829 (forward)	116598204..116598490, 116599390..116601829
RPA1CT	Chromosome 23 (CM066001.1)	70595335..70597774 (forward)	70594175..70594461, 70595335..70597774

All coordinates refer to the chromosome-level assembly of *N. tabacum* K326 (GenBank accession: AWOJ00000000; [71]) .

Supplementary Table 9. gRNA sequences and oligonucleotide pairs used for cloning.

			Insert					
			Forward primer introducing gRNA L			Reverse primer introducing gRNA R		
Plasmid	To generate mutant	Backbone	Oligo	gRNA	20-mer sequence	Oligo	gRNA	20-mer sequence
pEG004	*polq*$_{\Delta CDS}$*-3*	pJF1031	oEG003	c44	GGTATCCATTTGTGCAGAGA	oEG012	c271	GTGATTGCTGCAAAATGGAC
pEG007	*polq*$_{\Delta Hel}$*-4*	pJF1031	oEG003	c44	GGTATCCATTTGTGCAGAGA	oEG009	c182	GCAACAGCAGTAGCATTCAT
pEG017	*polq*$_{\Delta Pol}$*-1*	pEG001	oEG004	c214	GTGGATCCTCTGGCCTGATG	oEG011	c261	GCAGATAGAACTGAGATTGA
pEG019	*polq*$_{\Delta Pol}$*-2*	pEG001	oEG006	c251	GCACAGAGGTACACACTGCA	oEG013	c283	GAAGGGACGAAAGCGCTTCT
pEG021	*lig4-1*	pEG001	oEG015	c59	GTGACCCTAAGAGCCAGCTG	oEG018	c204	GGTAAAAGACGACAAACCAG
pEG022	*lig4-2*	pEG001	oEG016	c60	GCAGTAATGCATCTGCTGCG	oEG019	c205	GTAGGATTAGCATCTGCCTC
pEG025	*rpa1c* (not obtained)	pEG001	oEG022	c77	GAAAAGAGTTCTCCAGCTGA	oEG025	c176	GCTGCCAATAACATGACTCC
pEG028	*ku70*	pEG001	oEG028	c11	GAGAATTTACAGGACTTAAG	oEG031	c76	GAGCTGAACCCTCTAGGAAA
pEG030	*ku80*	pEG001	oEG033	c44-2	GATATCCTCTGCTGAGTGGG	oEG036	c62	GTCCTTGCCTCCATACACAT

Nucleotides in positions 2 to 20 are identical to the target sequence.

Supplementary Table 10. Sequences of oligonucleotides used as PCR primers in this study.

Name	Orientation	Sequence (5‘-3‘)	Purpose
oEG003	F	accaGGTCTCaatt**GGTATCCATTTGTGCAGAGA**gttttagagctagaaatagcaag	Cloning for CRISPR/Cas9 mutagenesis
oEG004	F	accaGGTCTCaatt**GTGGATCCTCTGGCCTGATG**gttttagagctagaaatagcaag	
oEG006	F	accaGGTCTCaatt**GCACAGAGGTACACACTGCA**gttttagagctagaaatagcaag	
oEG009	R	tggtGGTCTCtaaac**ATGAATGCTACTGCTGTTGC**aatctcttagtcgactctacc	
oEG011	R	tggtGGTCTCtaaac**TCAATCTCAGTTCTATCTGC**aatctcttagtcgactctacc	
oEG012	R	tggtGGTCTCtaaac**GTCCATTTTGCAGCAATCAC**aatctcttagtcgactctacc	
oEG013	R	tggtGGTCTCtaaac**AGAAGCGCTTTCGTCCCTTC**aatctcttagtcgactctacc	
oEG015	F	accaGGTCTCaatt**GTGACCCTAAGAGCCAGCTG**gttttagagctagaaatagcaag	
oEG016	F	accaGGTCTCaatt**GCAGTAATGCATCTGCTGCG**gttttagagctagaaatagcaag	
oEG018	R	tggtGGTCTCtaaac**CTGGTTTGTCGTCTTTTACC**aatctcttagtcgactctacc	
oEG019	R	tggtGGTCTCtaaac**GAGGCAGATGCTAATCCTAC**aatctcttagtcgactctacc	
oEG022	F	accaGGTCTCaatt**GAAAAGAGTTCTCCAGCTGA**gttttagagctagaaatagcaag	
oEG025	R	tggtGGTCTCtaaac**GGAGTCATGTTATTGGCAGC**aatctcttagtcgactctacc	
oEG028	F	accaGGTCTCaatt**GAGAATTTACAGGACTTAAG**gttttagagctagaaatagcaag	
oEG031	R	tggtGGTCTCtaaac**TTTCCTAGAGGGTTCAGCTC**aatctcttagtcgactctacc	
oEG033	F	accaGGTCTCaatt**GATATCCTCTGCTGAGTGGG**gttttagagctagaaatagcaag	
oEG036	R	tggtGGTCTCtaaac**ATGTGTATGGAGGCAAGGAC**aatctcttagtcgactctacc	
oEG095	F	TCCTCAAGGATAACCGCGAG	Genotyping of *cas9*
oEG298	R	CAGAGTGAGTGTCAGCACAA	
oEG229	R	AATCTGGATACGATCCCCGT	Genotyping of *LIG4* loci
oEG230	F	GGAATTGTCACCACATGGTG	
oEG231	R	AAGACACCAGAGCTAGGTCT	
oEG274	F	AGTTTTTGAGGTGGTATGTCATTG	
oEG052	F	GCTGAGATATTGATGTTACG	Genotyping of *POLQ* loci
oEG053	R	GGAGTTCATCAATCACTATG	
oEG058	F	GAGGAAGCAAGGCTTGCTGC	
oEG059	R	GACATCAAAGTAGAACTCTCC	
oEG062	F	GCGACTGTCAATGGAGGAAC	
oEG063	R	GAATTCACAGCTTGCCTCTG	
oEG086	F	CAGAGATTTGGTGGCTTGAG	
oEG088	F	GAATGTACTCTATGGGCATC	
oEG089	R	GGTTAGGTTCCTCCATTGAC	
oEG107	R	GATTCTTTCAGCAGCTTCATCTG	
oEG108	R	GAGCAACTTGTCGCATCTGATTC	
oEG361	R	CTTGATAATGTCTGCAGCTGAC	
oEG118	R	GTTGGTCTATCCAGATCCTC	Genotyping of *KU70* loci
oEG260	F	CTTCTTTCAACGCAAACTAGCTT	
oEG284	R	GCAGCTAGGTAATACTTCAGCTC	
oEG294	F	CTTCCCATTATCTGCAAGGTC	
oEG264	F	ATCTGAAGAGAAGTTTCCCACTC	Genotyping of *KU80* loci
oEG265	R	GAAGTAAAATGAATCAGGCTGCC	
oEG304	F	CTTCTAGGATTCACGGATGCTTC	
oEG305	R	CAGAAAGTGCAAGGATGGCC	
oEG381	F	GAGGCAGCGCGGCTATC	Genotyping of *nptII*
oEG382	R	GCGGTCCGCCACACCCA	

Bold; targeting sequence of the gRNA; underlined: sequences excised after BsaI cleavage (recognition site: GGTCTC).
