# Supplementary material for: Suppression of plastid-to-nucleus gene transfer by DNA double-strand break repair
Source: Nat Plants. 2025 May 16;11(6):1154–64. doi: 10.1038/s41477-025-02005-w (PMC12181080; doi:10.1038/s41477-025-02005-w)
Supplement: Supplementary file 2 — Reporting Summary [file 41477_2025_2005_MOESM2_ESM.pdf]

nature portfolio

Corresponding author(s): Enrique Gonzalez-Duran, Ralph Bock

Last updated by author(s): Apr 8, 2025

Reporting Summary

Nature Portfolio wishes to improve the reproducibility of the work that we publish. This form provides structure for consistency and transparency in reporting. For further information on Nature Portfolio policies, see our Editorial Policies and the Editorial Policy Checklist.

Statistics

For all statistical analyses, confirm that the following items are present in the figure legend, table legend, main text, or Methods section.

n/a	Confirmed	
☐	☒	The exact sample size (*n*) for each experimental group/condition, given as a discrete number and unit of measurement
☒	☐	A statement on whether measurements were taken from distinct samples or whether the same sample was measured repeatedly
☐	☒	The statistical test(s) used AND whether they are one- or two-sided<br>*Only common tests should be described solely by name; describe more complex techniques in the Methods section.*
☒	☐	A description of all covariates tested
☐	☒	A description of any assumptions or corrections, such as tests of normality and adjustment for multiple comparisons
☐	☒	A full description of the statistical parameters including central tendency (e.g. means) or other basic estimates (e.g. regression coefficient) AND variation (e.g. standard deviation) or associated estimates of uncertainty (e.g. confidence intervals)
☐	☒	For null hypothesis testing, the test statistic (e.g. *F*, *t*, *r*) with confidence intervals, effect sizes, degrees of freedom and *P* value noted<br>*Give P values as exact values whenever suitable.*
☒	☐	For Bayesian analysis, information on the choice of priors and Markov chain Monte Carlo settings
☒	☐	For hierarchical and complex designs, identification of the appropriate level for tests and full reporting of outcomes
☐	☒	Estimates of effect sizes (e.g. Cohen's *d*, Pearson's *r*), indicating how they were calculated

Our web collection on statistics for biologists contains articles on many of the points above.

Software and code

Policy information about availability of computer code

Data collection	No special software was used for data collection.
Data analysis	Statistical analysis and assembly of models was performed in R and Microsoft Excel 2021 (v. 2108). Save for one exception listed below, analysis was performed using R v.4.3.3 using the following packages: MASS v. 7.3-60.0.1, dplyr v. 1.1.4 , ggplot2 v. 3.5.1, ciTools v. 0.6.1, insight v. 0.20.0, rstudioapi v. 0.16.0, multcomp v. 1.4-25. The comparisons between slopes were performed using the phia package v.0.2-1, using R version 3.6.3. R code is deposited at https://github.com/egonzalezduran/modelsEGTdsbr.

For manuscripts utilizing custom algorithms or software that are central to the research but not yet described in published literature, software must be made available to editors and reviewers. We strongly encourage code deposition in a community repository (e.g. GitHub). See the Nature Portfolio guidelines for submitting code & software for further information.

Data

Policy information about availability of data

All manuscripts must include a data availability statement. This statement should provide the following information, where applicable:

- Accession codes, unique identifiers, or web links for publicly available datasets
- A description of any restrictions on data availability
- For clinical datasets or third party data, please ensure that the statement adheres to our policy

Data supporting the findings of this work are available within the paper, its Extended Data Figures manuscript and its Supplementary Information. Sequences from

Arabidopsis (AT1G49250.1, AT4G32700.2, AT5G45400.1, AT1G16970.1 and AT1G48050.1) are available through TAIR (https://www.arabidopsis.org/). Genomic sequences from Nicotiana (CM065996.1, CM065987.1, CM065979.1, CM066001.1, CM065985.1, CM065992.1, CM065997.1) are available at GenBank (https://www.ncbi.nlm.nih.gov/genbank/). Accession numbers are also provided in the relevant sections of the paper.

Research involving human participants, their data, or biological material

Policy information about studies with human participants or human data. See also policy information about sex, gender (identity/presentation), and sexual orientation and race, ethnicity and racism.

Reporting on sex and gender	N/A
Reporting on race, ethnicity, or other socially relevant groupings	N/A
Population characteristics	N/A
Recruitment	N/A
Ethics oversight	N/A

Note that full information on the approval of the study protocol must also be provided in the manuscript.

Field-specific reporting

Please select the one below that is the best fit for your research. If you are not sure, read the appropriate sections before making your selection.

☒ Life sciences ☐ Behavioural & social sciences ☐ Ecological, evolutionary & environmental sciences

For a reference copy of the document with all sections, see nature.com/documents/nr-reporting-summary-flat.pdf

Life sciences study design

All studies must disclose on these points even when the disclosure is negative.

Sample size	Minimal sample sizes were determined through power analysis. For somatic gene transfer experiments, the problem was modelled as a pairwise-comparison of counts of binary outcomes in the experiment between two genotypes: counts of leaf pieces that had produced EGT events, and those where no true events had been found (2x2 contingency table). Effect sizes were estimated by calculating Cohen's w, using the ES.w1() function in the pwr() package v.1.3-0 (https://cran.r-project.org/web/packages=pwr). For Experiment 1, power was set at 0.9 and type I error threshold (α)at 0.05: this threshold was corrected to account for multiple comparisons (Bonferroni). The sought effect size was that equivalent to a 30% reduction in FGT from the previously reported value for the RB98 genotype of 1 event/hlp (w = 0.03). Experiment 2 was designed to be confirmatory of the effects seen in Experiment 1, so power was set at the especially high value of 0.95, and the sought effects were of at least 50% of the difference found between RB98 and polqΔPol -1 in at the end of the first experiment (w = 0.05). Required sample sizes were calculated using the pwr.chisq.test() function in the pwr() package: 3944 and 1811 leaf pieces per genotype for Experiments 1 or 2, respectively. An excess of leaf pieces was used to ensure resilience to contaminations. The discovered linear time-dependence of gene transfer rate made it evident that comparisons at single time points were no longer appropriate: for this reason, the datasets were analyzed through generalized linear models. For pollen gene transfer experiments, sample sizes were established to detect a change in gene transfer rate from 1:16,000 (gene transfer rate in pollen in the original Huang et al.2003, Nature, 422(6927), 72–76) to 1:6,000 (w= 0.013..) through pairwise comparisons with power 0.95, and α=0.05, subsequently Bonferroni corrected (for six comparisons). Minimal sample size per group was determined to be 52,833 seeds.
Data exclusions	To select for independent events of somatic EGT, only the first event produced by a leaf piece was considered for further analysis. Totals of harvested candidate, EGT, or escape lines in somatic EGT experiments were cumulative (all lines were considered), but were not sampled at equal or consistent intervals. For balanced comparison between genotypes, only the last datapoints of non-overlapping 5-day windows were considered for statistical analysis. Datapoint grouping is detailed in Methods and Supplementary Table 2.
Replication	In somatic gene transfer experiments, the results proved reproducible after obtaining similar results in two large scale experiments for (i) the control Nt-RB98 and (ii) mutants generated independently. The pollen EGT experiment analyzed seeds from up to two harvests, each consisting of three pollen donors and up to three pollen recipient plants per genotype, resulting in dozens of cross-pollinated flowers. Reproducibility was shown by consistent results between independent mutants of the same class, and by the agreement between rates obtained in the independent harvests when applicable. Regeneration and bleomycin sensitivity tests were performed twice with similar results: replication was successful and representative images are shown. Experiments other than those mentioned here were repeated at least twice with similar results, with the exception of the procedures to obtain the individual DSBR mutants, which are in each case unique.
Randomization	For tissue culture experiments, leaf pieces were obtained from fully extended leaves. Plants of a genotype were produced in excess to obtain leaves for the experiment, and plants from which leaves were harvested were selected randomly. Plates in growth chambers were periodically shuffled randomly. In tissue culture experiments with only one growth condition (i.e., regeneration, EGT screen in tissue culture), comparisons were between groups and always with one group = one genotype: there was no need for covariate control. For experiments with more than a single growth condition (i.e., bleomycin concentrations), seeds were sown randomly.<br>Progenies to be genotyped were selected randomly, except in cases where genotype:phenotype linkage was examined. During pollination experiments, the pairing between pollen donors and recipient plants was random: once paired, maternal recipients received pollen only from

a single donor genotype. Since these experiments had only one growth condition, comparisons were between groups where always one group corresponded to one genotype of pollen donor: there was no need for covariate control. For all experiments other than those mentioned here, individual allocation was always random.

Blinding: Data collection and analysis was not blinded. There were no measurements or treatments that could significantly benefit from a blind design.

Reporting for specific materials, systems and methods

We require information from authors about some types of materials, experimental systems and methods used in many studies. Here, indicate whether each material, system or method listed is relevant to your study. If you are not sure if a list item applies to your research, read the appropriate section before selecting a response.

Materials & experimental systems

n/a	Involved in the study
☒	☐ Antibodies
☒	☐ Eukaryotic cell lines
☒	☐ Palaeontology and archaeology
☒	☐ Animals and other organisms
☒	☐ Clinical data
☒	☐ Dual use research of concern
☐	☒ Plants

Methods

n/a	Involved in the study
☒	☐ ChIP-seq
☒	☐ Flow cytometry
☒	☐ MRI-based neuroimaging

Dual use research of concern

Policy information about dual use research of concern

Hazards

Could the accidental, deliberate or reckless misuse of agents or technologies generated in the work, or the application of information presented in the manuscript, pose a threat to:

No	Yes
☒	☐ Public health
☒	☐ National security
☒	☐ Crops and/or livestock
☒	☐ Ecosystems
☒	☐ Any other significant area

Experiments of concern

Does the work involve any of these experiments of concern:

No	Yes
☒	☐ Demonstrate how to render a vaccine ineffective
☒	☐ Confer resistance to therapeutically useful antibiotics or antiviral agents
☒	☐ Enhance the virulence of a pathogen or render a nonpathogen virulent
☒	☐ Increase transmissibility of a pathogen
☒	☐ Alter the host range of a pathogen
☒	☐ Enable evasion of diagnostic/detection modalities
☒	☐ Enable the weaponization of a biological agent or toxin
☒	☐ Any other potentially harmful combination of experiments and agents

Plants

Seed stocks	All seed stocks and plant material used were generated in this work, with the exception of the transplastomic Nt-RB98 line, which was generated previously by our lab and was described in depth in Stegemann et al. (2003), PNAS, 100 (15), 8828-8833.
Novel plant genotypes	Novel tobacco DNA repair mutants were generated through genome editing using SpCas9 targeting the two homologs of each DSBR gene in N. tabacum with pairs of gRNAs. Genome editing constructs were assembled in binary vectors and delivered to plants by Agrobacterium-mediated transformation. A detailed account of the process (including gRNA sequences) can be found in Methods and Supplementary Materials. EGT lines containing novel insertions of plastid DNA in the nucleus were generated in the course of the gene transfer experiments.
Authentication	Novel mutant genotypes were confirmed by PCR followed by Sanger sequencing. Genotypes of plant materials were tracked through labelling of plants and seed stocks and use of an in-house database for laboratory work (LIMS). T-DNA insertions were detected by PCR of an amplicon within the cas9 sequence, which was segregated out before use in experiments. Genotypes at the DSBR loci were assessed by genotyping of plant progenies and confirmed when, in the double homozygous mutants, Sanger sequencing revealed a sequence corresponding to a single mutant allele per locus. Off-target minimization was considered during gRNA design, but frequency of off-targeting was not analyzed in planta. Potential secondary effects were assessed by comparison of independently generated mutants carrying comparable mutations. Phenotypes were assessed during vegetative growth and upon regeneration in tissue culture, while the sought loss-of-function of the target DNA repair factors was confirmed through bleomycin sensitivity assays.
