# Supplementary material for: Suppression of plastid-to-nucleus gene transfer by DNA double-strand break repair
Source: Nat Plants. 2025 May 16;11(6):1154–64. doi: 10.1038/s41477-025-02005-w (PMC12181080; doi:10.1038/s41477-025-02005-w)

Source Data: **Extended Data Fig. 5c.**

Unprocessed agarose gels Genotyping of LIG4 loci

Oligos oEG274 /oEG229 (Reaction 2)

Plants with wild-type like phenotype (n=48 + negative water control in last lane)

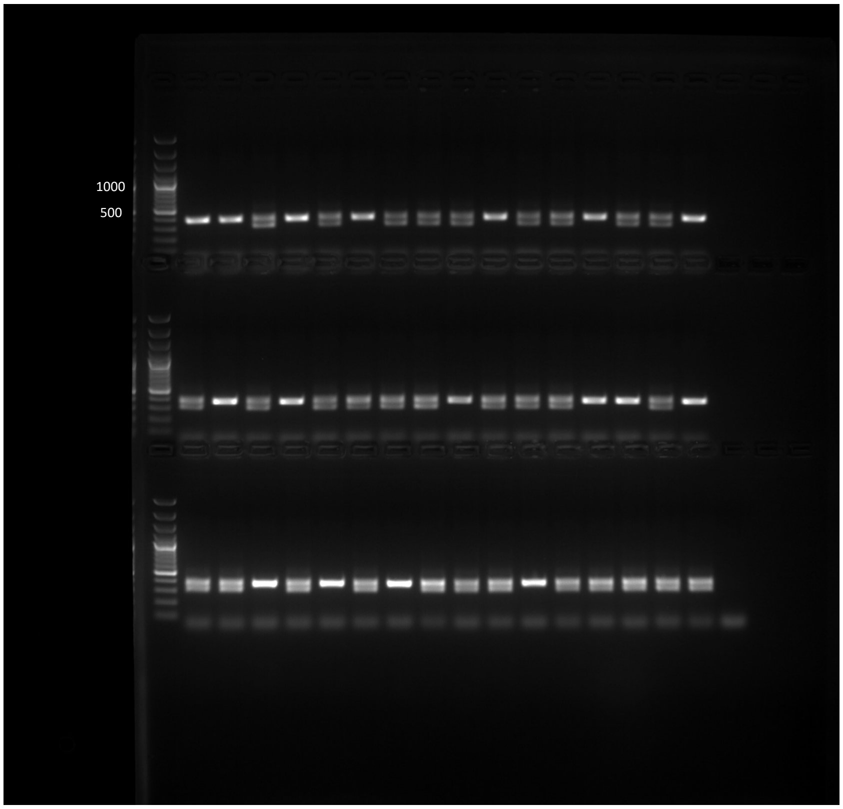

Plants with growth-arrested phenotype (n=46 + negative water control in last lane)

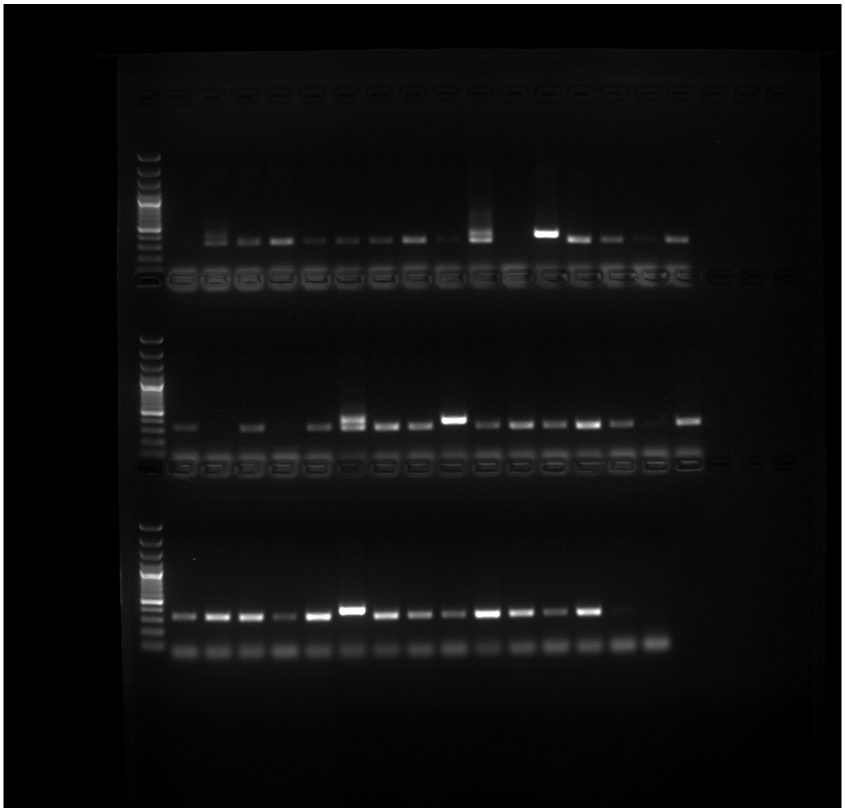

Supplement: Supplementary file 3 — Unprocessed agarose gels of the genotyping shown in Extended Data Fig. 5c. [file 41477_2025_2005_MOESM3_ESM.pdf]
